# Piezo1 induces Wnt7b^+^ astrocytes transformation to modulate glial scar stiffness and neuro-regeneration after stroke

**DOI:** 10.7150/thno.120838

**Published:** 2026-01-01

**Authors:** Shengju Wu, Wenjie Hao, Qian Suo, Qijie Lu, Ze Liu, Yang Qianbo Yao, Rubing Shi, Khan Haroon, Yuewei Chen, Xinfa Shao, Qinqin Wang, Chen Li, Qun Xu, Wanlu Li, Yaohui Tang, Guo-Yuan Yang, Zhijun Zhang

**Affiliations:** 1 Shanghai Jiao Tong Affiliated Sixth People's Hospital, School of Biomedical Engineering, Shanghai Jiao Tong University, Shanghai 200030, China.; 2 Shanghai Institute of Applied Physics, University of Chinese Academy of Sciences, Shanghai, 201204, China.; 3 Department of Health Management Center, Ren Ji Hospital, Shanghai Jiao Tong University School of Medicine, Shanghai 200127, China.; 4 Center for Single-Cell Omics, School of Public Health, Shanghai Jiao Tong University School of Medicine, Shanghai, China.

**Keywords:** glial scar stiffness, astrocyte subtypes, optogenetic regulation, neuroregeneration, ischemic stroke

## Abstract

**Background:** Reactive astrocytes form a chemical and mechanical glial scar that inhibits neuro-regeneration after stroke. Astrocyte heterogeneity is accompanied by changes in morphology and mechanical properties altering during scar formation after injury. This work aimed to elucidate the relationship between glial scar stiffness and astrocyte subtype transformation.

**Methods:** Astrocyte-specific archaerhodopsin-3 and channelrhodopsin-2 knock-in C57BL/6J mice underwent distal MCAO. Atomic force microscopy, ultrasound elastography and synchrotron radiation were used to determine changes in glial scar stiffness. A proteomic analysis of astrocyte subtypes was performed *ex vitro* using single-cell laser capture microdissection-MS. Furthermore, optogenetics was employed *in vivo* to reduce the glial scar stiffness, thereby facilitating neural regeneration following brain injury.

**Results:** Glial scar stiffness systematically increases following stroke and correlates with an increased number of Wnt7b^+^ fibrotic astrocytes. Furthermore, these results indicate that Piezo1 is the key regulator of astrocytic stiffness and anisotropy, which contributes to the glial scar stiffness in the peri-infarct area. The downregulation of Piezo1 expression promotes activation of the Wnt7b-Ca^2+^ nonclassical signaling pathway to modulate cytoskeletal reorganization. Finally, the specific optogenetic inhibition of Ca^2+^ signaling in astrocytes can effectively reduce glial scar stiffness by decreasing the proportion of Wn7b^+^ astrocytes, which further promotes neuro-regeneration and improves the recovery of motor function after ischemic stroke.

**Conclusions:** This study successfully revealed astrocyte subtype transformation as a key determinant of glial scar physical barrier formation after stroke and highlighted Piezo1 as a potential therapeutic target for modulating the mechanical microenvironment post-injury.

## Introduction

Stroke is a major global health burden and ranks as the second leading cause of death and the third leading cause of disability worldwide 1, 2. A glial scar, known as “border reactive astrocytes”, is a critical pathological feature after stroke. It represents a critical chemical and mechanical barrier that is formed mainly by reactive astrocytes and the extracellular matrix (ECM) around the injury site. It can not only separate damaged tissue from healthy tissue to avoid exacerbating inflammatory reactions but also inhibit neuro-regeneration in individuals with ischemic stroke 3. Although numerous studies have explored the chemical properties of glial scars, interventions aimed at either inhibiting glial scar formation or ablating established scars have not been shown to significantly enhance axonal regeneration. These findings underscore the dual protective and inhibitory roles of glial scars 4, 5. However, the mechanical properties of the glial scar, particularly stiffness, have received little attention. Research has shown that astrocytes near tissue injury sites exhibit a distinctive elongated morphology characterized by overlapping and interacting processes resembling those of fibrotic astrocytes after brain injury 6, whose distinct morphology may suggest varying degrees of stiffness 7. Furthermore, substrate stiffness is a crucial determinant of stem cell proliferation, differentiation, and migration 8-10, all of which are essential for endogenous and exogenous neuro-regeneration in the glial scar region after brain injury 11. This evidence inspired us to regulate the mechanical properties of the glial scar rather than its presence or absence to improve neurological recovery.

The heterogeneity of astrocytes after stroke is accompanied by morphological changes in addition to different functions. Cell morphology is influenced by both the intrinsic pressure of the cytoskeletal network and the extrinsic forces of the microenvironment, including neighboring cells or the ECM 12, 13. The balance between these intrinsic and extrinsic forces dictates the cell shape to maintain its normal function 7. The cytoskeleton serves as a crucial mechanical sensor of cellular force 14, 15. Noncanonical Wnt-Ca^2+^ or Wnt-PCP signaling is involved in regulating the actin cytoskeletal network, cell morphology, adhesion and migration in tumor cells 16, 17, suggesting that Wnt signaling regulates cell shape through the cytoskeleton, which in turn influences cell stiffness 18. The increased stiffness of reactive astrocytes correlates with the upregulation of intermediate filament expression after ischemia-reperfusion injury 19. Astrocytes may play a major role in the rigidity of the glial scar, which inhibits neuro-regeneration after injury and has been suggested as a therapeutic target for regeneration 20, 21. This evidence suggested that the distinctive morphologies of different astrocyte subtypes contribute to changes in brain stiffness after ischemic stroke. However, the cellular mechanism underlying the stiffness of glial scar remains unclear.

Increasing evidence has shown that various cell types transduce extracellular mechanical signals into intracellular signals through membrane mechanosensing channels, including piezoelectric ion channels within Piezo1 and Piezo2, to accomplish cellular morphological and functional transformations 22-25. In the brain, microglia sense the stiffness of Aβ fibrils via Piezo1 to induce their phagocytosis and compact Aβ plaques in individuals with Alzheimer's disease 24. Astrocytes can use Piezo1 to sense the mechanical environment of the brain and regulate neuro-regeneration and cognition in the adult brain 26. The ratio of neurons to astrocytes during neural stem cell (NSC) differentiation can be altered by substrate stiffness through Piezo1 27, which is also a crucial regulator of oligodendrocyte progenitor cell function in aging-related stiffness 28. However, whether Piezo1 is involved in the morphological changes in astrocytes contributing to altered stiffness and subsequent regeneration in the peri-infarct area after stroke is unknown.

We used GFAP-ChR2 and GFAP-Arch transgenic mice to manipulate astrocytic calcium signaling *in vivo*. ChR2 (channelrhodopsin-2) is a blue light (~473 nm)-activated cation channel that permits the influx of Na⁺ and Ca²⁺ ions, leading to membrane depolarization and increased intracellular calcium levels. Conversely, Arch (archaerhodopsin) is a green light (~560 nm)-driven proton pump that hyperpolarizes the cell membrane by exporting protons and suppressing voltage-gated calcium channels and intracellular calcium release, thereby reducing intracellular calcium signaling 29. Using these complementary optogenetic tools allows the bidirectional modulation of astrocytic calcium dynamics, enabling a detailed exploration of calcium-dependent astrocyte functions under ischemic conditions.

**Scheme 1:**
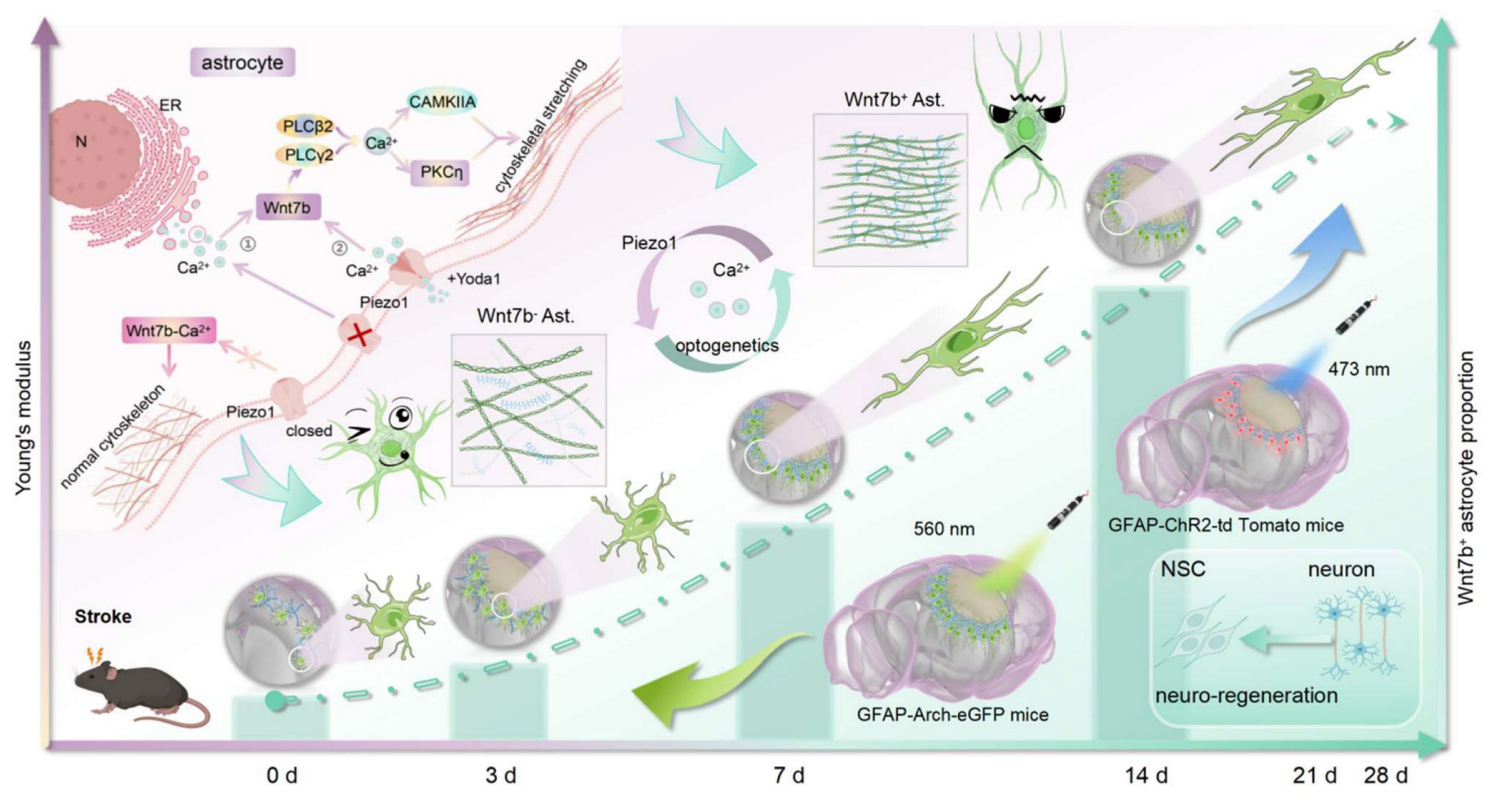
Using AFM, USE, and SR imaging, we demonstrated that glial scar stiffness progressively increased post-stroke, which positively correlated to the subtype of fibrous astrocytes. Integrated *in vitro* sequencing and *in vivo* single-cell microdissection spatial proteomics revealed that cytoskeletal reorganization was due to the increase of Wnt7b^+^ astrocytes. Furthermore, Piezo1 ion channels activated the non-canonical Wnt-Ca^2+^ signaling pathway through calcium influx, driving the transition Wnt7b^+^ astrocytes transformation. Optogenetic modulation of calcium signaling effectively reduced glial scar stiffness *in vivo*, promoted neuro-regeneration and significantly enhanced motor function recovery following ischemic stroke. AFM, atomic force microscopy; USE, ultrasound elastography; SR, synchrotron radiation; ChR2, channelrhodopsin 2; Arch, archaerhodopsin; N, nucleus; ER, endoplasmic reticulum.

In the present study, we combined atomic force microscopy (AFM), ultrasound elastography (USE), synchrotron radiation (SR) imaging and spatially resolved proteomics to explore the relationships between changes in glial scar stiffness and astrocyte subtypes after stroke. We found that a Piezo1-Ca^2+^-mediated increase in the proportion of Wnt7b^+^ astrocytes can regulate glial scar stiffness, which directs the NSC lineage choice during regeneration after ischemic stroke (**Scheme 1**).

## Materials and Methods

### Animal protocols

The animals were approved by the Institutional Animal Care and Use Committee (IACUC) of Shanghai Jiao Tong University, Shanghai, China (permission number: Bioethics 2023024). A total of 80 (22-25 g, 8 weeks) C57BL/6J mice (Vital River Laboratories, Beijing, China) were used. A total of 20 astrocyte-specific Arch knock-in C57BL/6J mice (GFAP-Cre-ERT2-Arch^+/+^, 22-25 g, 8 weeks) and 20 astrocyte specific CHR2 knock-in C57BL/6J mice (GFAP-Cre-ERT2-CHR2^+/+^, 22-25 g, 8 weeks) were used in this study. Arch knock-in mice (B6; 129S-*Gt(ROSA)26Sor^tm35.1(CAG-aop3/GFP)Hze^*/J, JAX stock #012735) and CHR2 knock-in mice (B6; Cg-Gt(ROSA)26Sor^tm27.1(CAG-COP4*H134R/tdTomato)Hze^/J, JAX stock #012567) were obtained from The Jackson Laboratory and used in combination with Cre-GFAP. All mice were maintained on a C57BL/6J background. The use of these models has been previously described 30. All the animals were housed in microisolator cages under a 12 h light/dark cycle (7:00 a.m. to 7:00 p.m.) with free access to food and water. The housing environment was maintained at 18-22 °C and 50%-60% relative humidity.

### Establishing a middle cerebral artery occlusion (MCAO) model

The mice were subjected to a distal MCAO model (**Figure S1F**) through 90 min of transient MCAO, as established previously 31. Briefly, we anesthetized the mice by administering 1.5%-2% isoflurane (RWD, Shenzhen, CN) through inhalation. For distal MCAO, a burr hole was drilled followed by craniotomy between the left eye and ear to expose the target artery. The MCA was occluded using an electric coagulator to interrupt blood flow while preventing damage to the brain parenchymal tissue. The dMCAO model was established without ligation of either the ipsilateral or contralateral common carotid artery (CCA). For transient MCAO, the carotid arteries were exposed through a midline neck incision. After dissection of the common, external, and internal carotid arteries, a silicon-coated 6-0 nylon suture (Covidien, Mansfield, MA) was inserted through the external carotid arteries and advanced retrograde along the internal carotid artery until it reached the origin of the middle cerebral artery. The suture was left in place for 90 min to induce occlusion. Subsequently, it was withdrawn to allow reperfusion, and the recovery of blood flow was assessed using laser Doppler flowmetry (Moon Instruments, Devon, UK) and laser speckle imaging (RWD, Shenzhen, CN). A successful occlusion was defined by an 85%-90% reduction in cerebral blood flow relative to the baseline.

### Examination of stiffness by atomic force microscopy (AFM)

Atomic force microscopy (AFM, Fast Scan Bio, Bruker) was used to assess Young's modulus of individual brain slices. We used a fast-scanning E-probe equipped with a gold-coated backing. The probe had the following specifications: a tip radius of 5 nm, a resonant frequency of 1 400 kHz, and a spring constant of 18 N/m. The experimental procedure began with the perfusion of the mouse heart using a pre-chilled PBS solution, followed by the rapid excision of brain tissue, which was subsequently sectioned into 1 mm thick slices. These slices were immersed in PBS to forestall softening. Subsequently, super glue was employed to securely affix the brain slices within 10 cm culture dishes. Deformation of the brain slices was meticulously monitored during the application of downward force by the probe microspheres. By analyzing the data derived from the force curve, we obtained the Young's modulus of the brain tissue. To ensure the precision of our measurements, we performed a minimum of 50 repetitions of test on the designated regions in each mouse brain slice.

Primary normal and fibrotic astrocytes were cultured in 24-well plates and maintained under physiological conditions. A 5 μm spherical probe was chosen for indentation tests to minimize cell damage. Target cells were localized using a light microscope, and their position and morphology were confirmed through pre-scanning. The indentation test was conducted in force curve mode, recording probe displacement and force signals. The data were fitted using the hertzian contact model to calculate the cells Young's modulus.

### Astrocyte anisotropy

Primary cultured normal astrocytes and fibrotic astrocytes were stained with phalloidin and imaged under a fluorescence microscope. The individual anisotropy coefficients of individual cells were shown using the aspect ratio 32. The ratio between the length and width of the cell was calculated using ImageJ software to characterize the pattern of the cytoskeletal distribution.

### Ultrasound elastography

The elastography of the high-end color Doppler ultrasound system Resona 7 by Mindray (Mindray, China) was performed for *in vivo* assessment of the elastic modulus of glial scars in adult C57BL/6 mice after ischemic stroke. Following the successful establishment of distal MCAO or transient MCAO models, we continuously monitored the elastic modulus values and strain ratios within distinct regions, namely, normal tissue areas, infarct core regions, and the vicinity of the injury (glial scar), at 7, 14, 21, and 28 days after ischemic stroke. Isoflurane anesthesia was administered to ensure uniform respiration in the mice during data acquisition, and the mice's cranial fur was shaved. Depending on the depth of the stroke-induced injury, a 20 MHz frequency ultrasound probe coupled with ultrasound gel was employed to induce transverse shear waves within the brain tissue, resulting in minimal mechanical deformation. After the acquisition of grayscale and elastic images, the delineation of a precise region of interest (ROI) delineation allowed us to derive absolute values.

### Decellularization of mouse brain tissue

Mouse brain tissue was decellularized using a modified protocol based on a previous study 33. Briefly, following transcardial perfusion with ice-cold 0.9% NaCl, mouse brains were rapidly harvested and sectioned into 2 mm coronal slices. For decellularization, the brain slices were placed in ddH_2_O for 7 h with shaking and then were immediately washed for 15 min using PBS. Individual sections were transferred to 24-well plates containing sodium deoxycholate (4%, m/v, Sigma, D7650) and incubated on a shaker for 14 h. Furthermore, the brain slices were again treated with DNase I (1 mg/mL, Sigma, DN25) in PBS with shaking for 1 h, followed by shaking and washing with ddH_2_O for 4 h. Finally, the brain slices were incubated with 3% (v/v) Triton X-100 for 2 h to disrupt the membrane and with DNase I (1 mg/mL) for 1 h. All critical steps involved shaking and washing with PBS for 15 min between steps to adequately remove the components from the previous step. After decellularization, the brain slices were snap-frozen in isopentane and sliced into 20 μm thick slices for HE and Masson staining for identification.

### Synchrotron radiation imaging

We precisely characterized the biomechanical properties of mouse brains following ischemic stroke employing the BL13HB beamline at the Shanghai Synchrotron Radiation Facility (SSRF) 34. To mitigate the potential thermal impact of X-rays on sample deformation during the imaging process, we implemented a dehydration pretreatment. Initially, mice were transcardially perfused with pre-chilled PBS to ensure optimal blood removal, followed by fixation (4% paraformaldehyde) at 4 °C. Subsequently, the dehydration treatment was conducted using alcohol solutions at concentrations of 50%, 60%, 70%, 80%, 90%, 95%, and 100%, with each concentration applied for a 24 h duration. Finally, the samples were immersed in salicylic acid for a period of 4-6 h, and subsequently subjected to drying in a 37 °C incubator until complete desiccation.

The sample was placed on a motorized rotation stage and secured with hot melt adhesive, situated 34 m away from the bending magnet source to conduct the CT scan. Following an assessment, we opted to use an 18 keV monochromatic X-ray beam for this experiment. Fluorescence imaging on the scintillator utilized 1.25× and 4× magnification microscope objectives and an SCMOS camera with a 6.5 µm pixel size, with the sample positioned 20 cm away from the detector. Initially, to observe the overall structure of mouse brain tissue, we utilized a 1.25× magnification objective with an effective pixel size of 5.2 µm and an exposure time of 50 ms. Additionally, we employed a 4× magnification objective to achieve enhanced clarity in the examination of glial scar structures, resulting in an effective pixel size of 1.625 µm and an exposure time of 200 ms. Under both conditions, we collected 1 080 projections over 180° for three-dimensional reconstruction. Subsequent to data acquisition, CT reconstruction was performed using PITRE.

### Primary fibrotic astrocyte isolation and culture

We used SD neonatal cortices (Vital River Laboratories, Beijing, China) as the source to obtain primary normal and fibrotic astrocytes. The entire process of primary cell isolation was executed in a sterile environment. Cortices were surgically separated using a scalpel and subsequently placed in pre-chilled Dulbecco's modified Eagle's medium (DMEM, HyClone, Logan, UT) with 1% P/S (penicillin and streptomycin) for further dissection. The brain tissue was maintained on ice and meninges and visible blood vessels were removed, ensuring the preservation of the cortical parenchyma. Afterward, we used 0.25% trypsin to enzymatically digest the isolated cells for a duration of 5-10 min, after which the digestion process was promptly terminated using DMEM with 10% FBS (Gibco, Carlsbad, NM). The resulting cell aggregates were uniformly dispersed into a single-cell suspension. Mixed glia was isolated through a 70 µm filter (Millipore) and uniformly cultured in the poly-D-lysine (PDL, Sigma, Aldrich)-coated T75 flasks. The culture medium for the mixed glia was changed every 3 days.

Confluent monolayers of normal astrocytes (> 90% confluence) were observed at the bottom of the culture flask, accompanied by the presence of microglia and oligodendrocyte precursor cells (OPCs) in the upper non-adherent or loosely attached layers. Given the differential adhesion properties of glial cell types (astrocytes > OPCs > microglia), we used sequential shaking at varying speeds and durations to selectively dislodge and isolate individual glial cell populations with high purity. Microglia were collected from the culture supernatant after shaking the T75 flasks at 180 rpm for 30 min. Subsequently, the original medium was replaced, and the cultures were shaken at 180 rpm for 16 h to dislodge OPCs, which were then collected from the fresh supernatant. The collected supernatant containing OPCs was centrifuged at 1,000 rpm for 5 min and seeded into 6-well plates. The differentiation of OPCs into fibrotic astrocytes was induced using the method established by Raff et al. 35, with minor optimization. Briefly, OPCs were cultured in DMEM/F-12 (1:1) with 15-20% fetal bovine serum (FBS) for 7 h to promote their differentiation toward a fibrotic astrocyte phenotype. To induce maturation of fibrotic astrocytes, the culture medium was switched to serum-free DMEM.

To induce fibrotic astrocyte differentiation, OPCs were maintained in DMEM/F-12 containing 20% fetal bovine serum (FBS) for 7 h. This serum-based induction protocol drives A2B5^+^ OPCs toward the type 2 astrocytic lineage under high-serum conditions. Elevated serum concentrations create a permissive environment enriched with cytokines (e.g., IL-6 and TGF-α), growth factors (e.g., EGF and bFGF), and bone morphogenetic proteins (BMPs), which synergistically activate the BMP-Smad-STAT3 signaling cascade, leading to the upregulation of astrocyte-specific markers such as GFAP. The astrocytes derived from this protocol exhibited an elongated, fibrotic-like morphology, consistent with the characteristics of reactive astrocytes.

### Transcriptomic sequencing

Following RNA extraction from astrocytes using TRIzol reagent (Invitrogen, USA) and quality assessment via an Agilent 2100 Bioanalyzer (Agilent Technologies, USA), sequencing libraries were constructed with the Illumina TruSeq Stranded mRNA LT Sample Prep Kit per the manufacturer's instructions. The transcriptomic sequencing and analytical services were provided by OE Biotech Co., Ltd. (Shanghai, China). A genome-wide transcriptomic analysis was conducted across four independent experiments. The differential gene expression analysis was performed using the DESeq (2012) R package with the functions estimate size factors and nbinom test. The criterion for significant differential expression was a *p* value < 0.05 coupled with a greater than 2-fold change. Subsequent functional annotation of these genes was performed via GO (Gene Ontology) and KEGG (Kyoto Encyclopedia of Genes and Genomes) pathway analysis implemented in the clusterProfiler R package, where enrichments with an FDR below 0.05 were retained. The GO terms and KEGG pathways were ranked in descending order according to their enrichment scores, and the top-ranked terms were visualized for presentation using the standard procedures, as previously described 36.

### Isolation of neural stem cells (NSCs)

The process for isolating NSCs closely resembled the method used to obtain mixed glia, with the exception that a 40 µm filter was employed, and the NSCs were maintained in a suspended spherical state. Approximately 3-4 day following the primary cell isolation, when the NSC spheres achieved uniform size and translucency, they were eligible for subculturing. Throughout the experimental procedures, the NSCs were typically used in the P2 generation and were identified through the expression of the NSC marker nestin.

### Laser capture microdissection-MS

The laser capture microdissection was completed using an MMI Cellcut Plus system (MMI, Zurich, Switzerland). Immunofluorescently labeled frozen sections were cut according to the guidelines of the instrument, which are briefly described below. The MMI membrane slide was placed over the glass slide and placed on the platform. The entire section was scanned under a 4× objective lens and then switched to a 20× objective to correct the tilt of the membrane slides. Different subtypes of astrocytes were manually circled with a stylus, which could be distinguished according to the fluorescence labeling. The samples were cut separately and collected in automatic mode. Next, we detected whether the target area attached to the tube cap to confirm successful collection and counted the number of cells. In this experiment, eight brain sections were used, approximately 50 cells were cut from each brain section, and then more than 400 cells were mixed into each sample for the proteomic analysis. In addition, 300 000 μm^2^ sections of the cortex, corpus callosum and striatum were cut from the brain to construct a self-established spectral library.

Proteins were extracted and digested as described previously with slight modifications^37^. Following supplementation with LCM-MTA lysis buffer, each sample was incubated in a water bath. Upon cooling to room temperature, centrifugation was carried out at 3,000 g for 30 sec. We then added chloroacetamide (0.5 M) and TCEP (0.5 M) to the samples, which were subsequently heated to 95 °C for 5 min to facilitate denaturation. After pre-washed magnetic beads were added, samples were shaken on a mixer at 1 200 rpm. Following removal of the supernatant on the magnetic rack, the beads were washed sequentially with 80% ethanol (twice) and 100% ethanol (once). The beads were resuspended in 0.25% trypsin (Meilunbio, MA0233-1, CN) in NH_4_HCO_3_ (100 mM) and digested overnight at 1 200 rpm at 37 °C. After centrifugation, the supernatants were collected into new tubes on a magnetic rack, mixed with 1% FA, and the peptide concentrations were quantified with a NanoDrop 1000 spectrophotometer (Thermo Fisher).

LC-MS/MS analysis was conducted on a Bruker timsTOF Pro system equipped with a trapped ion mobility spectrometer and a time-of-flight mass analyzer, interfaced with a NanoElute^®^ high-performance chromatograph (Bruker). Peptides were separated on a heated (60 °C) in-house packed C18 column (75 μm × 250 mm; 1.9 μm ReproSil-Pur beads, Dr. Maisch) using a 60-min linear gradient from 2% to 80% mobile phase B (0.1% formate in acetonitrile) at a flow rate of 300 nL/min. Mobile phase A was 0.1% formate in water. Mass spectrometric data were acquired in data-independent acquisition (DIA) mode with parallel accumulation-serial fragmentation (PASEF) enabled using default settings 38.

For database searches and data analysis, we processed the DIA label-free data with Spectronaut^®^ (v17; Biognosys). The public UniProt database was searched using a spectral library generated from 34 DIA runs. These runs encompassed a total of 300 000 μm² of tissue from the cortex, corpus callosum, and striatum, and the search was conducted with default parameters. Subsequently, the 28 DIA files were interrogated using this custom spectral library under standard search parameters.

We used R (version 4.2.2) to analyze the proteomics data. Subsequent analyses were based on median correction and log2 transformation. The Limma package was used for the analysis of differentially expressed protein, and the cutoff was set to a *p* value < 0.05, and a foldchange ≥2. Specific expression is defined as being expressed only in the target group and not in the other groups. The Mfuzz package was used for the time series analysis. We performed functional enrichment analyses for GO terms and KEGG pathways on the Metascape platform using the default parameters 39.

### Generation of polyacrylamide gels

We immersed glass siliconized coverslips with diameters of 14 mm (24-well plate), 20 mm (12-well plate), and 30 mm (6-well plate) in 75% alcohol, and subsequently cleansed them. These coverslips feature a positively charged surface, which facilitates cell adhesion. After sterilization, the coverslips were air-dried for use. We blended different concentrations of 40% (w/v) acrylamide (Arc, Sangon, 79-06-1, China), 2% (w/v) N, N-methylene-bis-acrylamide (Bis, Sangon, 110-26-9, China), and 1 mol 4-hydroxyethylpiperazine-1-ethanesulfonic acid (HEPES, Sigma, 7365-45-9, MO) to prepare hydrogels with varying degrees of stiffness, as outlined in **Table S1**. Subsequently, 10% (w/v) ammonium persulfate (APS, Sinopharm, 7727-54-0, China) and TEMED (0.004 g/mL, Thermo Fisher Scientific, 17919) were introduced into the acrylamide mixture at ratios of 1:100 and 1:1000, respectively. Next, we promptly dispensed 100 µL (12-well plate) of the gel solution onto a smooth mirror surface that had been previously treated with Gel Slick (Lonza Bioscience, 50640, Switzerland). The hydrophobic side of the coverslip was swiftly applied to cover the gel, taking care to prevent the formation of air bubbles. The gel was allowed to solidify under low-temperature conditions for 5-10 min, and then carefully peeled off using a scalpel and placed in the corresponding well of a plate. Following solidification, the hydrogel was immediately rinsed twice with a 50 mM HEPES solution (pH 8.5) to eliminate any excess precipitated solution. We activated the hydrogel and promoted the binding of proteins to the polyacrylamide substrates, using a sulfosuccinimidyl-6-(4-azido-2-nitrophenylamino)-hexanoate (0.2 mg/mL, sulfo-SANPAH, ProteoChem, 102568-43-4) activator that was dissolved in HEPES and subjected it to 365 nm UV light irradiation for 15 min or until the solution exhibited a darker hue. Subsequently, we conducted two additional washes using HEPES solution to mitigate the potential toxic effects of sulfo-SANPAH on cells. We used collagen type I (0.1 mg/mL, Corning Incorporated, 354236) to coat the hydrogel overnight to enhance cell adhesion.

### Drug treatment and calcium imaging

Yoda1 (MW = 355.3, C_13_H_8_C_l2_N_4_S_2_, purity > 98.00%, Glpbio, USA) is an activator of the mechanosensitive channel Piezo1. It was dissolved in DMSO and used at a working concentration of 5 μM. GsMTx4 (MW = 4101.89, C_185_H_279_N_49_O_45_S_6_, purity > 98.00%, Glpbio, USA) is an inhibitor of the mechanosensitive channel Piezo1. It was dissolved in DMSO and used at a working concentration of 2.5 μM. Thapsigargin (TG, MW = 650.75, C_34_H_50_O_12_, purity > 99.95%, MCE, USA) is a microsomal Ca^2+^-ATPase inhibitor. It was dissolved in DMSO and used at a working concentration of 5 nM. BAPTA AM (MW = 764.68, C_34_H_40_N_2_O_18_, purity > 99.68%, MCE, USA) is an intracellular calcium chelator that permeates cell membranes. It was dissolved in DMSO and used at a working concentration is 10 μM. We explored the effect of Piezo1 channels on calcium influx in astrocytes using Fluo-4 AM (Thermo Fisher Scientific, F14217, USA) to increase the calcium ion signals. The cells were added with 1 μM Fluo-4 AM in a carbon dioxide incubator for 30 min treatment, followed by three 10 min washes with PBS. Following the culture media replaced, the cells were allowed to equilibrate in the incubator for 20 min. We used Leica confocal fluorescence microscope to capture the images at 3 sec intervals over a 5 min period to monitor spontaneous calcium influx.

### Prediction of the structure of the Piezo1 and Wnt7b complex

We employed AlphaFold3, an advanced deep learning model for predicting the structures of protein complexes to demonstrate potential interaction between Piezo1 and Wnt7b. The analysis was performed with the default parameters, and the predicted structure of the complex was visualized and analyzed using PyMOL (version 2.5.4). Interactions between Piezo1 and Wnt7b were identified based on the atomic distance, with a threshold of <3.2 Å indicating a potential interaction. This distance criterion was chosen based on established standards for hydrogen bonding and van der Waals interactions in protein complexes. The predicted interaction sites were further validated by analyzing residue contact maps and surface electrostatic potential.

### Coimmunoprecipitation (Co-IP) assay

We harvested primary astrocytes, treated them with Yoda1 (5 µM) to establish the experimental group, and subsequently prepared protein lysates. The total protein concentration of the lysates was adjusted to 1 μg/mL for downstream analyses. We used protein A/G magnetic beads (MCE, HY-K0202-1, 2 μm, USA) conjugated to antibodies against Wnt7b (Santa Cruz, sc-365459, USA) and Piezo1 (Thermo Fisher Scientific, 28511-1-AP, USA). Pretreated magnetic beads (50 µL) were conjugated to 400 µL diluted antibody, washed thoroughly, and then incubated with 400 µL antigen sample to form the antigen-antibody-magnetic bead complex. Subsequently, 50 µL 1× SDS-PAGE loading buffer was added to the complex, mixed, and heated at 95 ℃ for 5 min to fully denature and elute the proteins. Western blot analysis, employing 10% and 7.5% separating gels for Wnt7b and Piezo1 detection respectively, verified the presence of both proteins in the collected supernatant.

### Laser stimulation

Astrocytes in GFAP-Cre-ChR2-tdTomato and GFAP-Cre-Arch-GFP transgenic mice from the laser stimulation group were subjected to activation using a pulsed 473 nm (blue) or 560 nm (yellow) laser (Shanghai Laser & Optics Century Co., Ltd., BL473T3-050FC). This stimulation was administered for 15 min sessions on post-operative days 3, 4, 5, and 6 following distal MCAO.

### BrdU and EdU administration and proliferation assays

We added 10 μM BrdU (Beyotime, ST1056) to assess NSC proliferation on matrix gels with different stiffnesses. The immunostaining protocol is described below.

EdU (Beyotime, ST067) was administered via intraperitoneal injection at 50 mg/kg/ day from 3 to 6 days after distal MCAO in all light stimulation groups to assess astrocyte proliferation, neuro-regeneration and angiogenesis study. We performed the BeyoClick™ EdU Cell Proliferation Kit with Alexa Fluor 555 (Meilunbio, MA0425-1, CN) and Alexa Fluor 488 (Meilunbio, MA0424-1, CN) to assess cell proliferation according to the manufacturer's instructions.

### NSC transplantation

We isolated the primary GFP-NSCs from the cortex of GFP-labeled newborn mice and cultured as stable spheroids for 3 days before they were transplanted into the glial scar area after ischemic stroke. For the *in vivo* assessment of differentiation, we transplanted 200 000 cells into the peri-infarct cortical region (AP: +1.0 mm, ML: -3.5 mm, DV: +1.0 mm). Fourteen days after the original transplantation, the brain tissue was cryosectioned, and the differentiation capacity of GFP-NSCs was assessed using DCX and GFAP immunostaining.

### Immunofluorescence staining

Brain tissue samples were frozen and sectioned into 25 µm slices, which were then placed in an antifreeze solution (50% PBS, 20% glycerol, 30% ethylene glycol, v/v%) and stored at -20 °C. We used 0.4% PFA to fix the brain sections and cells for 10 min, followed by membrane permeabilization with 0.3% Triton X-100 (Sigma, St Louis, MO) for an additional 10 min. Subsequently, a blocking step was performed using 2-5% BSA (bovine serum albumin, Gibco, MA) for 1 h at room temperature. The samples were incubated with primary antibodies more than 16 h at 4 °C. The following antibodies were used: goat anti-GFAP (1:400, ab53554, Abcam), guinea pig anti-GFAP (1:200, OB-PGP055-02), rabbit anti-Wnt7b (1:200, DF9042, Affinity), rabbit anti-Piezo1 (1:100, DF12083, Affinity), Phalloidin-iFluor (300 tests, ab176753, Abcam), mouse anti-Tuj-1(1:200, MAB1637, Millipore), rabbit anti-DCX (1:200, ab18723, Abcam), mouse anti-Nestin (1:100, MAB353, Millipore), mouse anti-CSPG (1:200, C8035-100UL, Sigma), rabbit anti-Collagen I (1:200, ab21286, Abcam), rat anti-BrdU (1:200, ab6326, Abcam), goat anti-CD31 (1:300, AF3628, R&D), rabbit anti-S100β (1:200, EP1576Y, Abcam), mouse anti-SMI31 (1:200, 801601 , Biolegend), rat anti-MBP (1:200, ab-7349), mouse anti-MAG (1:200, 66709-1-1g), and mouse anti-PDGFRA (1:200, Santa Cruze). After three rinses with PBS, the samples were incubated with the relative secondary antibodies at 37 °C for 1 h. Subsequently, they were mounted using DAPI-containing mounting media (P0131, Beyotime) for storage. We used an inverted confocal fluorescence microscope (Leica, Wetzlar, Germany) to acquire the fluorescence images.

### siRNA transfection and real-time PCR analysis

We plated a total of 1×10^5^ cells in each well of a 24-well plate for transfection, combining 20 nM siRNA (OBio. Technology, Shanghai) with 50 μL of Opti-MEM (Meilunbio, Dalian, China) in one tube. In another new tube, 50 μL Opti-MEM and 1 μL Lipofectamine^TM^ 3000 (Thermo Fisher, L3000001, Scientific USA) were combined. Following dilution, the Lipofectamine 3000 reagent was mixed with the RNA and incubated at RT for 20 min with gentle agitation. Afterward, the resulting mixture was added to each well for 10 min, followed by the addition of the corresponding culture media. The following siRNA sequences were employed for gene interference: siRNA-Piezo1, 5'- TCGGCGCTTGCTAGAACTTCA-3'; siRNA-Trpm7, 5'- TCATGAAGGTGCTGGGTAA-3'; siRNA-Wnt7b, 5'- GCAUGAAGCUGGAAUGUAATT-3', 5'- UUACAUUCCAGCUUCAUGCTT-3'. Forty-eight hours after the siRNAs had been successfully transfected, real-time PCR was performed to assess the transfection efficiency, and detailed primer sequences are listed in **Table S2**.

### Western blot analysis

Primary cultured astrocytes were washed with PBS, after which proteins were extracted using pre-chilled lysis buffer. The sample protein concentrations were determined using a bicinchoninic acid (BCA) kit (Meilunbio, Dalian, China). Equal amounts of each protein sample were loaded onto 10% (w/v) and 7.5% (w/v) sodium dodecyl sulfate-polyacrylamide gel electrophoresis (SDS-PAGE) gels, followed by electrophoretic separation and subsequent transfer onto PVDF membranes (polyvinylidene difluoride, Millipore). The membranes were incubated more than 16 h at 4 ℃ with the following first antibodies: Wnt7b (1:500, sc-365459, Santa Cruz), PLCβ2 (1:1000, DF4721, Affinity), PLCγ2 (1:1000, WL02220, Wanlebio), Rac (1:1000, WL02851, Wanlebio), Cdc42 (1:1000, WL01165, Wanlebio), CaMKIIa (1:1000, WL03453, Wanlebio), CaMK IV (1:1000, WL04335), PKCη (1:1000, HA722389, HUABIO), and β-actin (1:3000, 66009, Invitrogen). The membranes were incubated for 1 h at RT with HRP-conjugated secondary antibodies, selected based on the species origin of the respective primary antibodies. The protein signals were visualized by an incubation with enhanced chemiluminescence (ECL) substrate (Pierce, Rockford, IL, USA), and the resulting chemiluminescence signals were quantified using ImageJ software.

### Statistical analysis

1. used one-way ANOVA followed by Dunnett's test to analyze the data, unless indicated otherwise. Statistical significance was defined as a *p* value < 0.05. All data are expressed as the mean ± SD. Statistical analyses and image presentation were performed using GraphPad Prism, version 9.0.

## Results

### The increase in glial scar stiffness over time after ischemic stroke attributes to cell type

**Figure 1:**
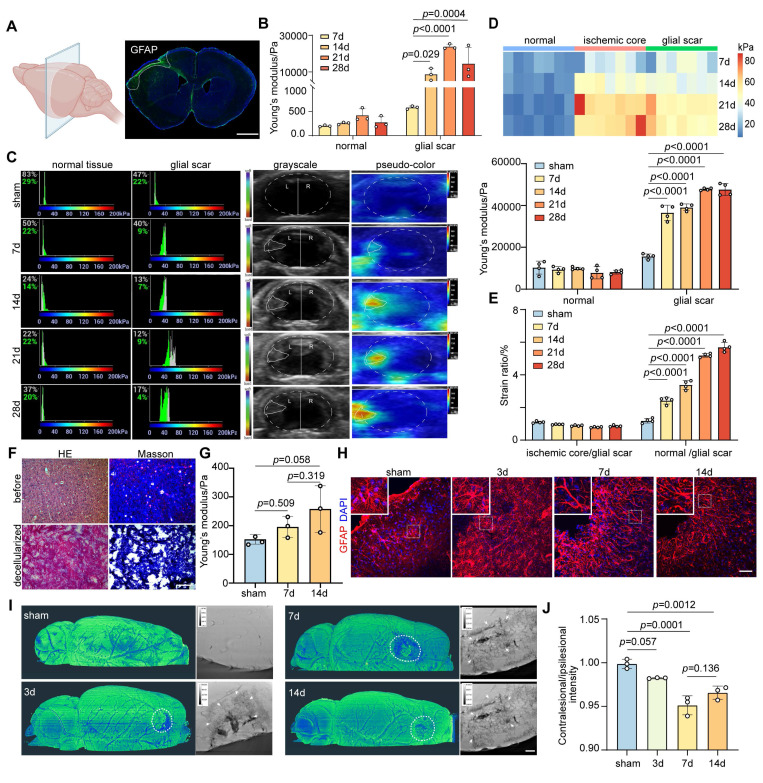
** Glial scar stiffness increased over time after distal middle cerebral artery occlusion. A** Schematic diagram of brain slices from mice subjected to distal middle cerebral artery occlusion (MCAO). The fluorescent imaging represents the coronal plane of brain at 28 days after distal MCAO. Scale bar = 1 mm. **B** Young's modulus of normal tissue and glial scar at 7, 14, 21, 28 days after distal MCAO mice using atomic force microscopy. Data compared to the time point at 7 days (n = 3 mice per group). **C** Representative images of stiffness detected by ultrasound elastography in normal tissue and glial scar of sham operated mice, 7, 14, 21, 28 days after distal MCAO mice, respectively, highlighting the glial scar region in grayscale (left column) and pseudo-color image (right column). Solid white lines represent brain parenchyma and glial scar outlines. The left panel displays the percentage of the maximum possible stiffness value within the stiffness range of the target area (The gray percentage represents the center area of the ROI, while the green percentage represents the shell of the ROI). **D** and **E** Statistical analysis showing changes in Young's modulus (**D**) and strain ratio (**E**) over time after MCAO (n = 4 mice per group). **F** HE and Masson staining of mouse brain tissue before and after decellularization. Scale bar = 100 μm. **G** Young's modulus of sham, 7 and 14 days after distal MCAO decellularization using AFM (n = 3 mice per group). **H** Representative immunostaining images of GFAP (red) in sham operated and 3, 7, 14 days after distal MCAO groups, highlighting the development of glial scar tissue. Scale bar = 50 μm. **I** and **J** Representative images of Synchrotron radiation phase-contrast imaging (**I**) and the intensity ratio comparison (**J**) between the contralateral and ipsilateral region in sham operated mice and the peri-infarct area at 3, 7, 14 days after MCAO (n = 3 mice). White arrow representing the accumulated distribution of cells in glial scar. Scale = 100 μm. Using one-way ANOVA followed by Dunnett's test with data compared to the sham operated group (**B, D, E, G, J**). All data are represented as mean ± SD.

We employed distal middle cerebral artery occlusion (distal MCAO) and transient middle cerebral artery occlusion (transient MCAO) models to analyze the mechanical properties of glial scar in the coronal and sagittal planes, respectively (**Figure 1A and Figure S1A**). *Ex vivo* examinations of Young's modulus in the glial scar region were conducted using AFM at 7, 14, 21, and 28 days after ischemic stroke. Young's modulus gradually increased starting from the formation of the gelatinous scar on Day 7 (633 ± 104 Pa), and reached its peak on Day 21 (22 400 ± 301.5 Pa). Conversely, the stiffness of the normal tissue did not change significantly after ischemic stroke (**Figure 1B**). We simultaneously employed USE to monitor stiffness changes of glial scar *in vivo* to mitigate the potential reduction of stiffness associated with *ex vivo* detection in brain slices. The results revealed that the trends in Young's modulus and strain ratio were consistent with the *ex vivo* findings (**Figure 1C-E and Figure S1B-E**).

The stiffness of tissue is governed by a combination of constituent cell types and ECM components 12. We examined the expression trends of the main ECM components CSPG and Col I after ischemic stroke to evaluate the contribution of ECM and cell type to increased stiffness of glial scar and detected a weak correlation between the ECM components expression and the glial scar stiffness (**Figure S2A-F**). Furthermore, we decellularized brain slices from mice in the sham group and distal MCAO group at 7 and 14 days after surgery, respectively (**Figure S2G**). HE and Masson's trichrome staining of the brain tissue before and after decellularization revealed the absence of cellular components and the preservation of ECM components (**Figure 1F**). Young's modulus of the glial scar region in cortex was 152.5 ± 16.86 Pa, 195.7 ± 36.06 Pa and 257.9 ± 80.76 Pa after decellularization in the sham group and ischemic stroke group at 7 and 14 days, respectively (**Figure 1G**). Interestingly, the absolute Young's modulus values of the decellularized brain slices were significantly lower than those of the normal brain slices at the same time point (the value of the sham group was 218 ± 37 Pa, the value of 7 days was 633 ± 104 Pa, and the value of 14 days was 7 822 ± 3 156 Pa without decellularization, as shown in **Figure 1B**), suggesting that cellular components contribute substantially to the change in glial scar stiffness, whereas changes in ECM may play a relatively minor role. Following ischemic stroke, the gradual formation of the glial scar is attributed primarily to gliosis, with astrocytes constituting the main cellular component. On Day 7, we noted a transition in the astrocyte morphology, that was characterized by a fibrous appearance with a reduced cytosolic volume and elongated branches (**Figure 1H**). Additionally, using synchrotron phase-contrast imaging, we quantified the transmittance via grayscale values to elucidate the microstructure of glial scar after ischemic stroke. The grayscale value in the peri-infarct area was lower than that of sham group, which reached the lowest point at 7 days after ischemic stroke, revealing the most severe cellular aggregation in the glial scar region (**Figure S3 and Figure 1I-J**), and suggesting that the increase in glial scar stiffness after ischemic stroke could be due to changes in the cellular subtype.

### Wnt7b^+^ astrocytes contribute to the increase in glial scar stiffness after ischemic stroke

**Figure 2:**
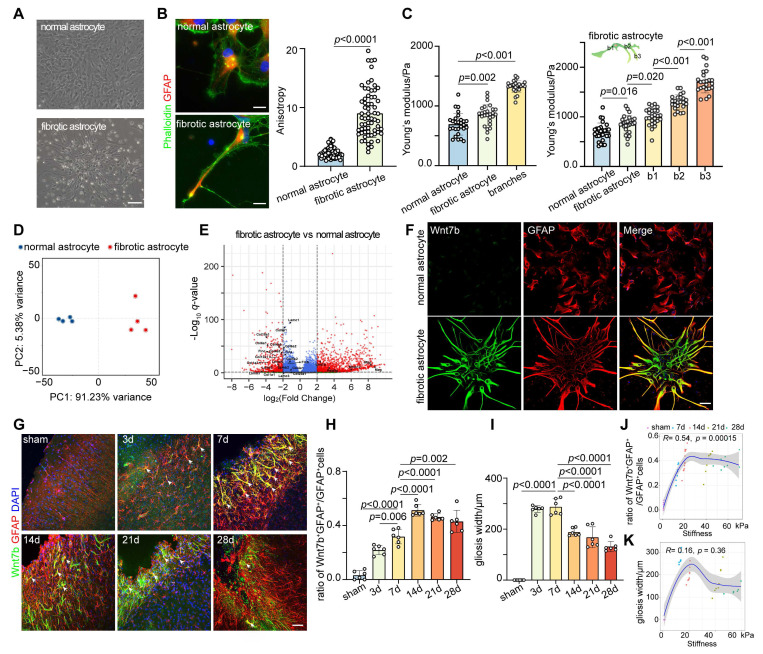
** Wnt7b^+^ astrocytes contribute to the increase of glial scar stiffness after ischemic stroke. A** Bright-field microscopy images showing normal astrocytes and fibrotic astrocyte. Scale bar = 50 μm. **B** Representative images and quantification of cytoskeleton distribution in normal and fibrotic astrocytes, phalloidin (green), GFAP (red). Scale bar = 100 μm. Anisotropy was represented in terms of individual cell aspect ratios, with each dot representing one cell. Using two-tailed unpaired Student's test (n = 66 cells). **C** *In vitro* assessment of Young's modulus for cytoplasmic region of normal and fibrotic astrocytes, and for branches (b1, b2, b3) of fibrotic astrocytes by AFM. b1, b2, and b3 refer to the first, second, and third branches of fibrotic astrocytes, respectively (n = 24 cells). Statistical analysis was conducted using one-way ANOVA followed by Dunnett's test. **D** Principal component analysis (PCA) plot demonstrating clustering patterns of gene expression profiles between normal and fibrotic astrocytes. **E** Volcano plot illustrating differentially expressed genes (upregulated on the right, downregulated on the left) between normal and fibrotic astrocytes, *p* < 0.05. **F** Immunostaining images of normal and fibrotic astrocytes labeled with DAPI (blue), Wnt7b (green), GFAP (red). Scale bar = 50 μm. **G** Representative immunostaining images showing Wnt7b^+^GFAP^+^ astrocytes in the glial scar region at sham operated and 3, 7, 14, 21, 28 days after distal MCAO group. Wnt7b (green), GFAP (red) and DAPI (blue). Scale bar = 50 μm. **H** and **I** Quantification of Wn7b^+^GFAP^+^/GFAP^+^ cells ratio (**H**) and the width of gliosis (**I**) in the glial scar region (n = 6 mice per group). Using one-way ANOVA followed by Dunnett's test. **J** and **K** Correlation analysis depicting the relationship between glial scar stiffness and the ratio of Wn7b^+^GFAP^+^/GFAP^+^ cells (**J**), and between glial scar stiffness and gliosis width (**K**) over time following distal MCAO. All data are represented as mean ± SD.

Therefore, we speculated that the stiffness of the glial scar was related to the fibrotic astrocyte subtype after stroke. We cultured oligodendrocyte precursor cell-derived fibrotic astrocytes and normal astrocytes from cortex to clarify the presence of fibrotic astrocyte subtypes within the glial scar region (**Figure 2A**). We first characterized the cytoskeletal distribution of these two cell types using phalloidin staining and found that the anisotropy (aspect ratio) of fibrotic astrocytes was significantly greater than that of normal astrocytes (**Figure 2B**). The Young's modulus of the cytoplasm and cytoskeletal branches was measured by AFM to assess the influence of orientation-dependent structural characteristics on mechanical properties. Young's modulus of fibrotic astrocytes (850.0 ± 177 Pa) was higher than that of normal astrocytes (694.0 ± 182 Pa), and the stiffness of the branches (b1 994 ± 173 Pa, b2 1 299 ± 147 Pa, b3 1 711 ± 228 Pa) increased progressively with increasing distance from the cytoplasm (**Figure 2C**). We performed transcriptomic sequencing and validation experiments to clarify the molecular characteristics of fibrotic astrocytes (**Figure 2D-E**). Based on the OPC origin of fibrotic astrocytes and after removing the interference of inflammation-related genes (**Tables S3 and S4**), the RNA-sequencing results indicated that Pdgfra, Mag, Mbp, and Wnt7b were the top markers to distinguish fibrotic astrocytes from normal astrocytes. Since Wnt7b is a key gene involved in extracellular matrix and cytoskeletal regulation and the expression of the other genes in fibrotic astrocytes was not as prominent (**Figure S4**), we identified Wnt7b as a potential marker for fibrotic astrocytes. Further immunostaining of brain slices revealed that Wnt7b was effectively co-labeled with GFAP in fibrotic astrocytes but minimally expressed in normal astrocytes, indicating that Wnt7b could be a candidate marker gene for fibrotic astrocytes (**Figure 2F**). Next, we validated *in vivo* the presence of fibrotic astrocytes within the glial scar and found that the fraction of fibrotic astrocytes (Wnt7b^+^GFAP^+^ cells) among total reactive astrocytes (GFAP^+^ cells) increased over time following ischemic stroke (**Figure 2G-H**), which was significantly correlated with the stiffness of the glial scar (**Figure 2J**). Despite the progressive decrease in the width of glial scar as reactive astrocyte barrier tightened over time, the width and the stiffness were not correlated (**Figure 2I, K**). The results indicated that the progressive increase in the proportion of Wnt7b^+^ fibrotic astrocytes over time post-stroke is a primary driver for the increased stiffness of glial scar.

### The increase of Wnt7b^+^ astrocytes was due to cytoskeletal reorganization after stroke

**Figure 3:**
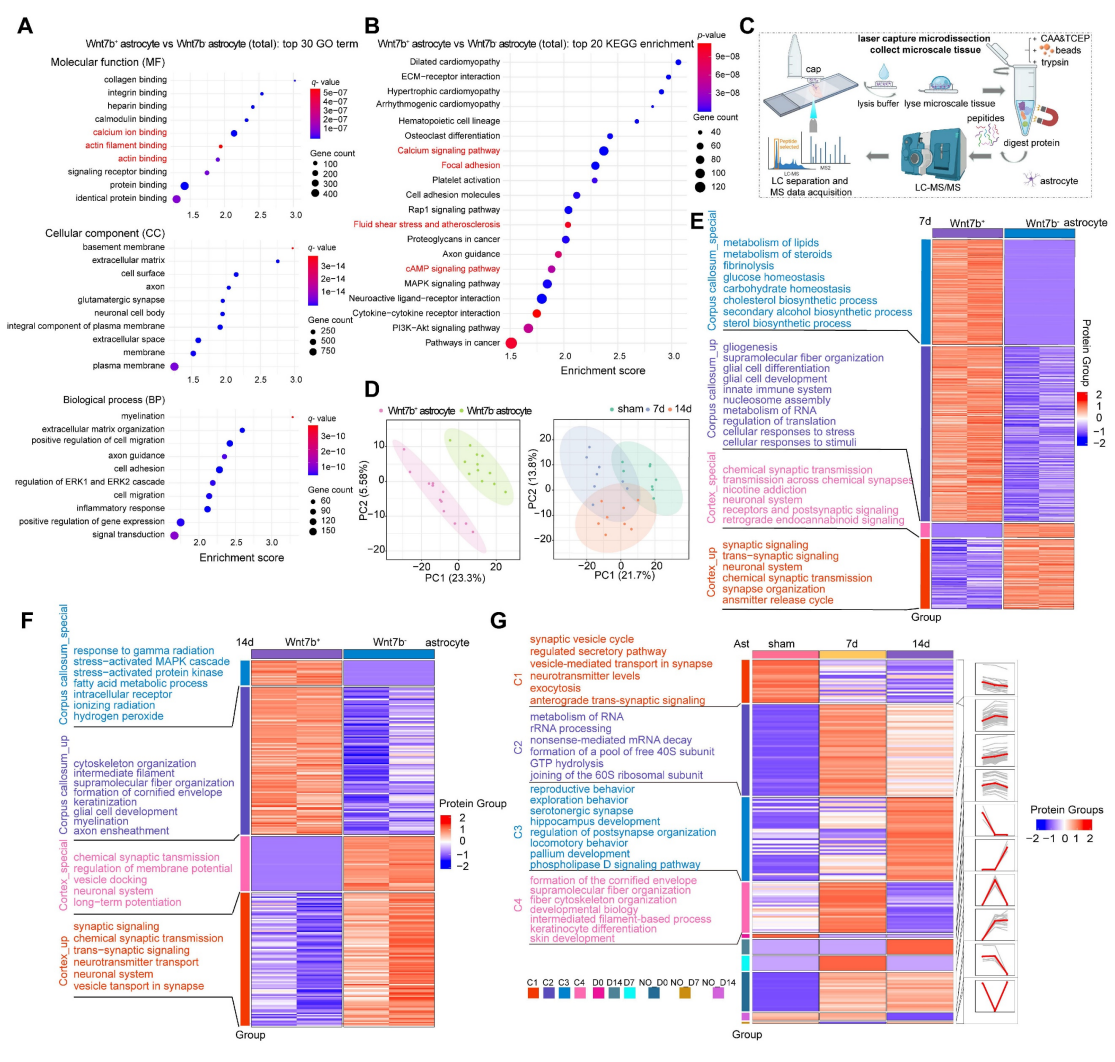
** The increase of Wnt7b^+^ astrocytes stiffness was due to cytoskeleton reorganization after stroke. A** Bubble plot displaying the top 30 enriched GO terms in fibrotic astrocytes compared to normal astrocytes (Wnt7b^-^ astrocyte). (*p* < 0.05, fold change > 2). **B** Bubble plot displaying the top 20 enriched KEGG pathways in fibrotic astrocytes compared to Wnt7b^-^ astrocyte. (*p* < 0.05, fold change > 2). **C** Schematic diagram of the laser capture microdissection spatial proteomics process. **D** Principal component analysis (PCA) comparing Wnt7b^-^ astrocyte and Wnt7b^+^ astrocytes at 0, 7, and 14 days after ischemic stroke respectively. Each data point represents an immunofluorescence brain slice from different animal brains. In the comparison of time line in sham, 7 and 14 days after stroke (n = 8 brain slices with 400 cells per group). **E** and **F** Heat map displaying functional enrichment analysis of Wnt7b^-^ astrocytes and Wnt7b^+^ astrocytes at 7 days (**E**) and 14 days (**F**) after ischemic stroke (sample n = 2 indicates two independent repeated measurements). Enrich groups were categorized as follows: corpus callosum_special, corpus callosum_up, cortex_special, and cortex_up. **G** Heat map illustrating functional enrichment of astrocytes in sham operated mice and 7, 14 days after ischemic stroke. Different categories (C1-C4, D0, D14, D7, NO-D0, NO-D7, and NO-D14) were based on expression trends over time.

GO terms and KEGG pathways enrichment analyses were performed to further explore the reason for the increase in the stiffness of Wnt7b^+^ fibrotic astrocytes, which was reflected in a macroscopic increase in glial scar stiffness, and the results demonstrated that primary Wnt7b^+^ fibrotic astrocytes were enriched in processes related to actin filament binding, calcium ion binding, focal adhesion, and calcium signaling pathways compared to Wnt7b^-^ astrocytes (**Figure 3A-B**). Given the distinct features of Wnt7b^+^ fibrotic and Wnt7b^-^ astrocytes, we employed a microspatial resolved proteomics method suitable for tissue slices produced using laser capture microdissection (LCM), termed LCM magnetic trace analysis (LCM-MTA), which can significantly improve the sensitivity, recovery rate, and integrity compared with conventional proteomic approaches 37, 40-42. Therefore, we performed a proteomic analysis of Wnt7b^+^ fibrotic and Wnt7b^-^ astrocytes in the peri-infarct area using LCM-MTA, considering their spatiotemporal distribution patterns prior to and after ischemic stroke (**Figure 3C**). We performed immunofluorescence staining of brain slices from similar anatomical locations from sham, day 7 and 14 post stroke groups and collected Wnt7b^-^ astrocytes and Wnt7b^+^ fibrotic astrocytes cells from the peri-infarct area. A minimum of 50 cells were collected from each brain slice (**Figure 3D**). Cross-axis comparisons at the same time point revealed that compared with Wnt7b^-^ astrocytes, Wnt7b^+^ astrocytes were enriched in functions related to gliogenesis, supramolecular fiber organization, and intermediate filament cytoskeleton organization (**Figure 3E-F**). We categorized proteins according to their temporal expression patterns into Cluster1 (C1), Cluster2 (C2), Cluster3 (C3) and Cluster4 (C4). Among them, C4 showed the most pronounced enrichment of cytoskeletal and intermediate filament-associated proteins on day 7 after ischemic stroke, which may explain the rapid increase in Young's modulus of glial scar from 7 days to 14 days after stroke (**Figure 3G**). These results suggested that the disparity in morphology between Wnt7b^+^ astrocytes and Wnt7b^-^ astrocytes was influenced by the cytoskeleton and supramolecular fiber organization.

### Piezo1 regulates astrocyte cytoskeletal reorganization through Wnt7b-Ca^2+^ signaling pathway

**Figure 4:**
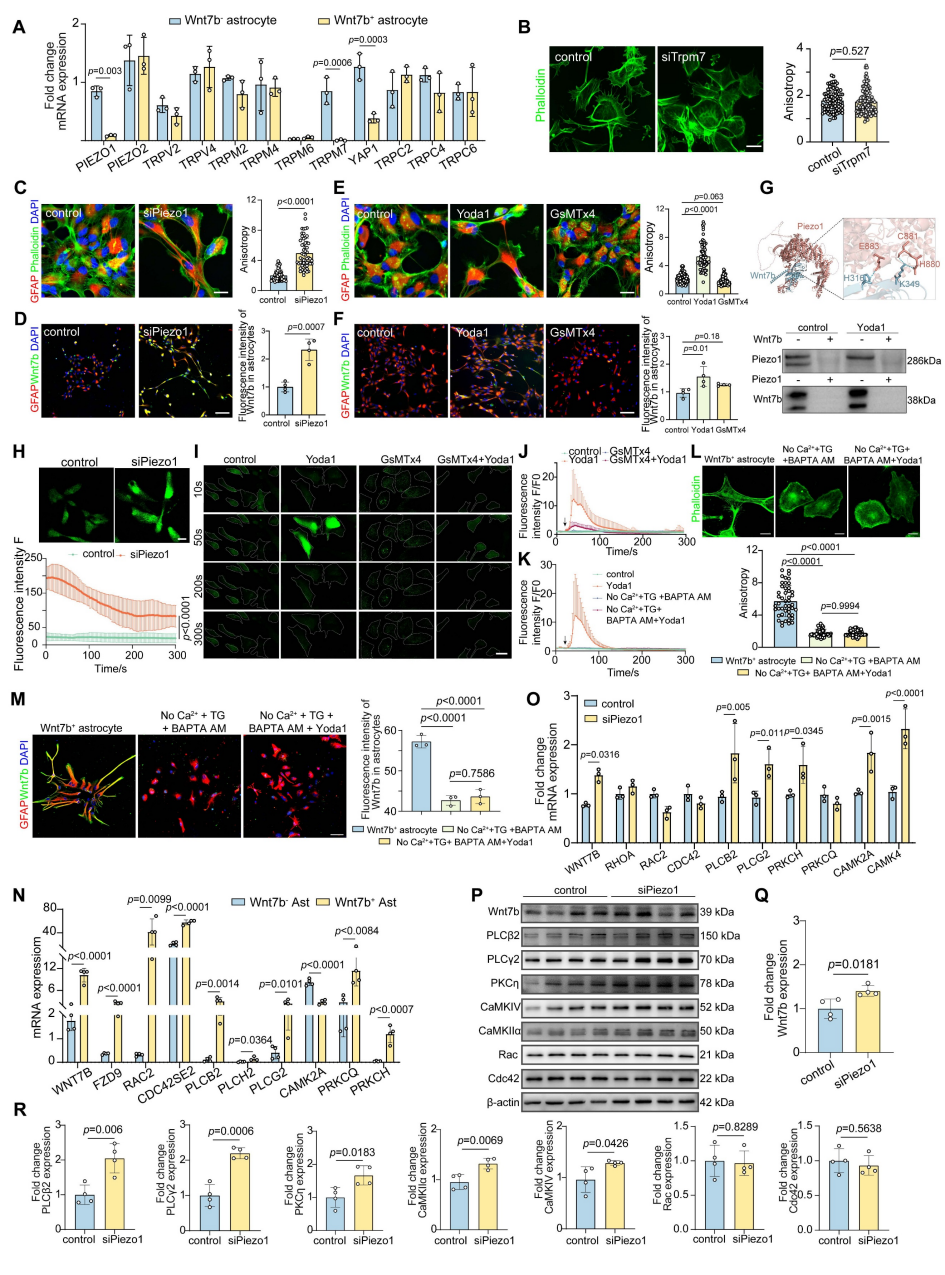
** Piezo1 regulates astrocyte cytoskeleton reorganization through Wnt7b-Ca^2+^ signal pathway. A** Real-time PCR analysis of stress related genes between normal and Wnt7b^+^ astrocyte (n = 3 independent primary astrocytes cultures). GAPDH was used as an internal control. **B** Representative immunostaining images demonstrating siRNA-mediated interference with Trpm7 expression and quantitative analysis of anisotropy in primary astrocytes culture. Scale bar = 50 μm. Statistical significance was determined using two-tailed unpaired Student's test (n = 104 cells). **C** Representative immunostaining images showing siRNA interference with Piezo1 expression in primary astrocytes and quantitative analysis of anisotropy in control and siRNA Piezo1groups *in vitro*. Scale bar = 100 μm. Phalloidin (green) and GFAP (red) visualization followed (n = 52 cells). **D** Representative immunostaining images of Wnt7b (green) in astrocytes (GFAP, red) following siRNA interference with Piezo1 expression. Scale bar = 50 μm. And quantification of fluorescence intensity of Wnt7b in astrocytes (n = 4 slices per group). **E** Representative immunostaining images of primary astrocytes with the Piezo1 activator Yoda1 (5 μM) and the inhibitor GsMTx4 (2.5 μM) for 24 h compared to the DMSO control group. Phalloidin (green) and GFAP (red) with quantitative anisotropy analysis. Scale bar = 100 μm (n = 72 cells). **F** Representative immunostaining images of Wnt7b (green) in astrocytes (GFAP, red) following of Piezo1 activity by Yoda1 and GsMTx4. Scale bar = 100 μm. And quantification of Wnt7b fluorescence intensity in astrocytes (n = 4 slices per group). **G** Structural docking and co-immunoprecipitation analysis of Wnt7b and Piezo1 interaction. The upper panel represents the predicted binding sites of Wnt7b (blue) and Piezo1 (red) obtained through AlphaFold3-based 1:1 dynamic docking. H316: Histidine 316, K349: Lysine 349, H880: Histidine 880, C881: Cysteine 881, E883: Glutamic acid 883. The lower panel shows the results of the Co-IP experiment, where Piezo1 and Wnt7b were analyzed under control and Yoda1-treated conditions. **H** Representative images of Ca^2+^ imaging in Fluo-4 AM pretreated astrocytes following control and siPiezo1 treatment. Scale bar = 10 μm. **I** Representative images of Ca^2+^ imaging in Fluo-4 AM pretreated astrocytes following Piezo1 activation or inhibition. Different treatment groups included DMSO (control), Yoda1 (5 μM), GsMTx4 (2.5 μM), and GsMTx4+Yoda1 (with GsMTx4 pre-treatment for 30 min). Scale bar = 10 μm. **J** Quantification of fluorescence intensity F/F0 representing the real-time Ca^2+^ changes relative to baseline (F0) in DMSO (control), Yoda1 (5 μM), GsMTx4 (2.5 μM), and GsMTx4+Yoda1 treatment groups. **K** Quantification of fluorescence intensity F/F0 representing the real-time Ca^2+^ changes relative to baseline (F0) in control, Yoda1 (5 μM), no Ca^2+^-free DMEM+TG (5 nM) + BAPTA AM (10 μM) and no Ca^2+^-free DMEM+TG (5 nM) +BAPTA AM (10 μM) +Yoda1 treatment groups. **L** Representative immunostaining images of cytoskeleton expression and quantitative anisotropy analysis after Ca^2+^-free DMEM+TG (5 nM) +BAPTA AM (10 μM) and Ca^2+^-free DMEM+TG (5 nM) +BAPTA AM (10 μM) +Yoda1 treatment in astrocyte culture. Scale bar = 50 μm in control groupand 10 μm in treatment group. Statistical significance was determined using two-tailed unpaired Student's test (n = 47 cells). **M** Representative immunostaining images of Wnt7b (green) in astrocytes (GFAP, red) following Ca^2+^-free DMEM+TG (5 nM) +BAPTA AM (10 μM) and Ca^2+^-free DMEM+TG (5 nM) +BAPTA AM (10 μM) +Yoda1 treatment. Scale bar = 50 μm. And quantification of Wnt7b fluorescence intensity in astrocytes (n = 3 slices per group). **N** Real-time PCR analysis of Wnt pathway related genes between Wnt7b^-^ and Wnt7b^+^ astrocytes from RNA-Seq (n = 4 independent primary astrocytes cultures). GAPDH was used as an internal control. **O** Real-time PCR analysis of Wnt pathway related genes between control and siPiezo1 astrocytes (n = 3 independent primary astrocytes cultures). GAPDH was used as an internal control. **P-R** Representative Western blot images and quantification of analysis of Wnt signaling pathway proteins (n = 4 independent primary astrocytes cultures). Using T-test and all data are represented as mean ± SD.

The KEGG enrichment analysis revealed that Wnt7b^+^ astrocytes exhibited an increased expression of proteins associated with fluid shear stress, atherosclerosis, and the cAMP signaling pathway (**Figure 3B**). Additionally, LCM-MTA analysis indicated that these cells were linked to cellular responses to stress, the stress-activated MAPK cascade, and stress-activated protein kinase signaling cascade-related pathways. Furthermore, protein biological processes such as cytoskeletal reorganization, super large molecule fiber reorganization, and intermediate filaments were significantly enriched in the corpus callosum_specific group and corpus callosum_up group of Wnt7b^+^ astrocytes (**Figure 3E-F**). These results suggested the potential involvement of stress-associated mechanosensitive ion channels in morphological changes. Subsequently, we examined the mRNA levels of several mechanosensitive ion channels potentially involved in regulating the morphological features of astrocytes and found that the Trpm7 and Piezo1 ion channels may play crucial roles in normal Wnt7b^-^ astrocytes and fibrotic Wnt7b^+^ astrocytes (**Figure 4A**). To further verify which one make more contribution to the morphological changes, we performed siRNA experiment for Trpm7 and Piezo1 ion channels separately *in vitro*. The results showed that knockdown of Trpm7 in Wnt7b^-^ astrocytes did not influence the anisotropy coefficient (**Figure 4B**), whereas the knockdown of Piezo1 ion channel led to an increase in the anisotropy coefficient compared with that in the control group (**Figure 4C**). Furthermore, the morphology of Wnt7b^-^ astrocytes was fibrotic after Yoda1 (a Piezo1 activator) and GsMTx4 (a Piezo1 inhibitor) treatment (**Figure 4E**). Notably, decreasing the expression and altering the activity of Piezo1 ion channels also increased the expression of Wnt7b in astrocytes (**Figure 4D-F**), suggesting the possibility of interconversion between Wnt7b^-^ astrocytes and Wnt7b^+^ fibrotic astrocytes after ischemic stroke and that the Piezo1 ion channel in potentially involved in this process. Moreover, under the same experimental conditions, activation or inhibition of Piezo1 in Wnt7b^+^ fibrotic astrocytes did not significantly alter either the cell morphology or Wnt7b expression levels, which may be attributable to the relatively low expression of Piezo1 in Wnt7b⁺ astrocytes (**Figure S5**). Interestingly, we found that Piezo1 was predominantly centrally distributed in the cytoplasm of Wnt7b^-^ astrocytes, particularly in the nuclear membrane, but was mostly located in the branches of Wnt7b^+^ fibrotic astrocytes (**Figure S6A-C**), suggesting that Piezo1 substantially contributes to changes in astrocyte morphology and stiffness.

To elucidate the interactions between Piezo1 and Wnt7b proteins, we employed AlphaFold3 to predict the complex structure of the two proteins at a 1:1 molecular ratio. The analysis revealed key amino acid interaction sites, including H316 and K349 in Wnt7b, and E883, C881, and N880 in Piezo1. Moreover, we used coimmunoprecipitation to verify the lack of a significant direct interaction between Piezo1 and Wnt7b (**Figure 4G**). Furthermore, because the knockdown of Piezo1 expression and activation of its channel activity significantly increased calcium influx signaling in astrocytes (**Figure 4H-J**), we hypothesized that Piezo1 indirectly regulates Wnt7b expression through Ca^2+^ signaling, thereby modulating cytoskeletal reorganization. Subsequently, we used calcium-free medium supplemented with thapsigargin (TG, 5 nM) and BAPTA AM (10 µM) to completely chelate calcium in astrocytes. The optimal concentrations of TG and BAPTA AM were determined to ensure that they did not cause cell damage (**Figure S7A-D**). This intervention abolished the calcium signal (**Figure S7E and Figure 4K**). Moreover, calcium depletion in astrocytes significantly reduced the anisotropy of Wnt7b^+^ fibrotic astrocytes and downregulated Wnt7b expression, but Yoda1 activation did not reverse these changes (**Figure 4L-M**). These findings suggest that Piezo1-mediated regulation of Wnt7b^+^ fibrotic astrocyte transformation is calcium-dependent.

Moreover, compared with those in Wnt7b^-^ astrocytes, genes associated with the Wnt-PCP and Wnt-Ca^2+^ pathways were significantly upregulated in Wnt7b^+^ astrocytes (**Figure 4N**), suggesting that increased Wnt7b expression may subsequently regulate cytoskeletal reorganization through the noncanonical Wnt pathway. Following astrocyte intracellular and extracellular calcium depletion, the expression of genes related to the Wnt-Ca^2+^ signaling pathway was significantly decreased at the mRNA level but remained unaltered by Yoda1 treatment. In contrast, the expression of Wnt-PCP related genes did not change significantly (**Figure S7F**). Subsequently, we verified the mRNA and protein levels in astrocytes after Piezo1 knockdown. Piezo1 knockdown significantly upregulated the expression of Wnt-Ca^2+^ pathway-associated proteins, including Wnt7b, PLCβ2, PLCγ2, PKCη, CaMKIIα, and CaMKIV, whereas the expression of the Wnt-PCP pathway proteins, Rac and Cdc42 did not change significantly (**Figure 4O-R**). We next investigated the molecular mechanism underlying the effects of Wnt7b on astrocyte morphology. The overexpression of Wnt7b in Wnt7b⁻ astrocytes significantly increased cellular anisotropy and Wnt7b expression, accompanied by the marked upregulation of the expression of Wnt-Ca²⁺ signaling pathway genes (PLCB2, PRKCH, PRKCQ, CAMK2A and CAMK4) but not the expression of Wnt-PCP genes (RAC2 and CDC42). Conversely, Wnt7b knockdown in Wnt7b^+^ astrocytes decreased cellular anisotropy and downregulated the expression genes related to Wnt-Ca²⁺ signaling genes (**Figure S8**). These results indicate that Wnt7b drives the astrocyte phenotypic transition through Ca²⁺-dependent pathways.

Overall, the expression and activity of the Piezo1 ion channel modulated the astrocyte morphology and structure. The disruption of Piezo1 ion channel homeostasis activated the noncanonical Wnt7b-Ca^2+^ signaling pathway, which modulated the reorganization of the cytoskeleton and supramolecular fibers, thereby regulating cellular morphology and function in response to external environmental stimuli.

### Astrocyte-specific photo-stimulation modulates Wnt7b^+^ astrocytes proportion via Ca^2+^ thereby altering glial scar stiffness

**Figure 5:**
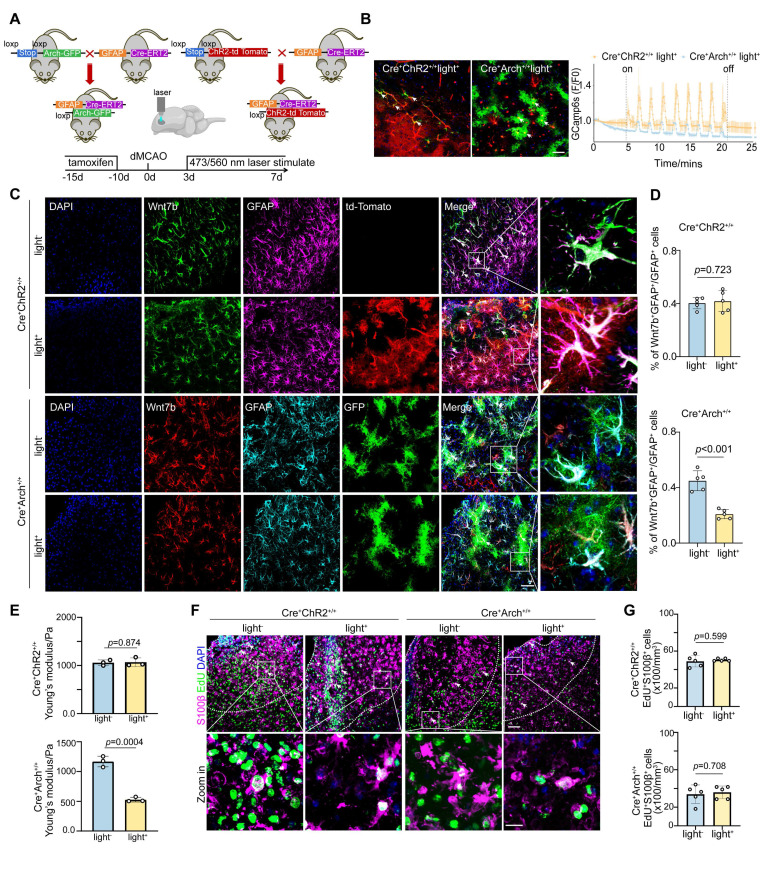
** Astrocyte-specific photo-stimulation alters glial scar stiffness by Ca^2+^ signaling mediated Wnt7b^+^ astrocytes transformation. A** Schematic diagram illustrating the construction of photo-stimulated transgenic mice and *in vivo* experiments set up. **B** Trend plots representing changes of astrocytes calcium imaging *in vivo* detected by 15 min stimulation with 473 nm or 560 nm laser light. The white arrows in fluorescence representative images denoting astrocytes infected by the pAAV-GCamp6s virus in ChR2 and Arch mice. Scale bar = 50 μm. **C** Representative immunostaining images showing Wnt7b^+^ (green) GFAP^+^ (purple) cells following photo-activation (ChR2, td-tomato, red) and Wnt7b^+^ (red) GFAP^+^ (turquoise) cells following photo-inhibition (Arch, GFP, green). Scale bar = 50 μm. **D** Quantification of Wnt7b^+^GFAP^+^/GFAP^+^ ratio in ChR2 photo-activation or Arch photo-inhibition mice (n = 5 mice per group). **E** Young's modulus of glial scar was measured by AFM in mice subjected to ChR2 photo-activation or Arch photoinhibition at 7 days after distal MCAO (n = 3 mice per group). **F** Representative immunostaining images illustrating EdU^+^ (green) S100β^+^ (purple) cells following photo-activation or photo-inhibition in mice. Dashed lines showed the glial scar area. Scale bar = 50 μm and 10 μm in zoom images. **G** Quantification of EdU^+^S100β^+^ cell number in mice subjected to ChR2 photo-activation or Arch photo-inhibition mice (n = 5 mice per group). Two-tailed unpaired Student's test was used in (**D**, **E**, **G**) panels. All data are represented as mean ± SD.

Given the pivotal role of Ca^2+^ signaling in the regulation of the fibrotic astrocyte morphology by Piezo1 (**Figure 4**), we investigated whether the Ca^2+^ level within astrocytes in glial scar region directly modulates the proportion of Wnt7b^+^ astrocytes after stroke. We verified this effect by generating astrocyte-specific channel rhodopsin 2 (ChR2) transgenic mice by crossbreeding GFAP-cre-ERT2 mice with floxed-stop-ChR2-td Tomato mice, and astrocyte-specific archaerhodopsin (Arch) transgenic mice by crossbreeding GFAP-cre-ERT2 mice with floxed-stop-Arch-GFP mice (**Figure 5A**). After tamoxifen treatment, efficient expression of ChR2-td Tomato^+^ and Arch-GFP^+^ cells was observed in cells throughout the entire brain slice (**Figure S9A**). We injected *p*AAV-GfaABC1D-GCaMP6s, an astrocyte-specific green shifted Ca^2+^ indicator, into the GFAP-ChR2 mice and GFAP-Arch mice (AP: +1.0 mm, ML: -3.5 mm, DV: +1.0 mm). After 3 weeks, the activity of GCaMP6s-expressing astrocytes was recorded by delivering 473 nm blue light or 560 nm yellow light stimulation, confirming altered Ca^2+^ signaling in the astrocytes after optogenetic stimulation. Compared with GFAP-ChR2 mice, GFAP-Arch mice significantly reduced calcium signaling within astrocytes in the glial scar (**Figure 5B**).

After successfully establishing the optogenetic mouse model, we assessed the proportion of Wnt7b^+^ astrocytes on Day 7 following ischemic stroke. The findings revealed that photo-inhibition in GFAP-Arch mice led to a significant reduction in the proportion of Wnt7b^+^ astrocytes, whereas photo-activation in GFAP-ChR2 mice did not affect the proportion of these cells (**Figure 5C-D**). Surprisingly, under GFAP-Arch photo-inhibitory conditions, Young's modulus of glial scar decreased, which indicated that the stiffness of glial scar could be modulated *in vivo*. Conversely, GFAP-ChR2 photo-activation mice did not alter Young's modulus (**Figure 5E**). In addition, to exclude whether the reduction in glial scar stiffness induced by photo-inhibition was caused by astrocyte proliferation decrease, we examined the effect of photo-inhibition on astrocyte proliferation by administering EdU starting Day 3 after ischemic stroke. Results showed that neither astrocyte-specific photo-activation nor astrocyte specific photo-inhibition impacted the proliferative capacity of astrocytes (**Figure 5F-G**), which suggested that photo-inhibition reduced the stiffness of glial scar because of the decrease in the proportion of Wnt7b^+^ astrocytes.

### The decrease of glial scar stiffness improves neuro-regeneration and behavior recovery after stroke

**Figure 6:**
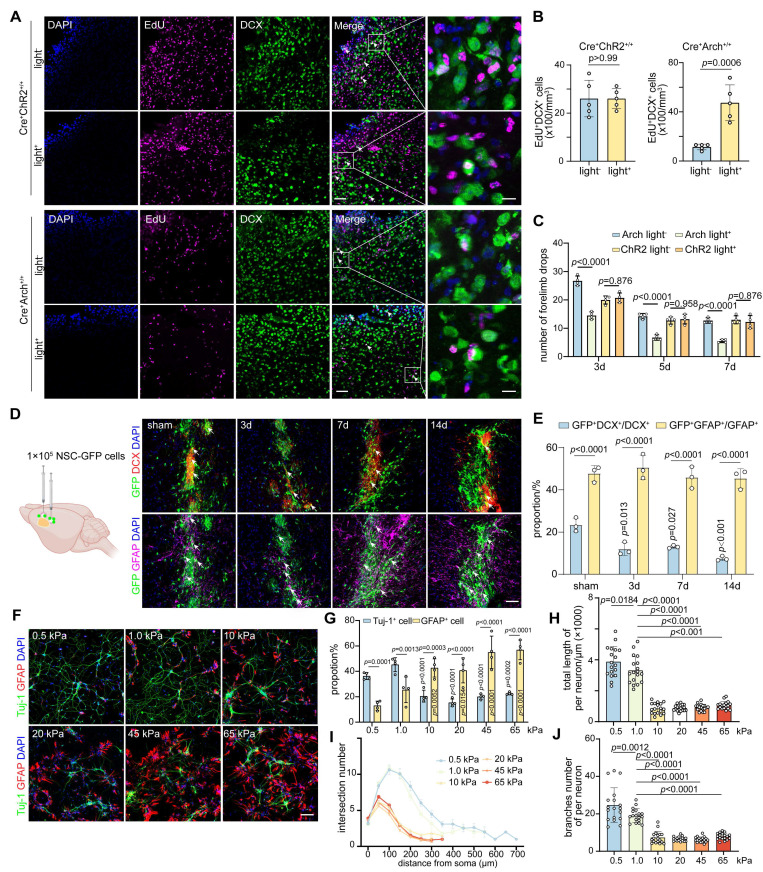
** The decrease of glial scar stiffness modulated by Wnt7b^+^ astrocytes transformation improves neuro-regeneration and behavior recovery after stroke. A** Representative immunostaining images of EdU^+^ (purple) DCX^+^ (green) cells following ChR2 photo-activation or Arch photo-inhibition in mice. Scale bar = 50 μm. **B** Quantification of EdU^+^DCX^+^ cell number in ChR2 photo-activation or Arch photo-inhibition mice (n = 5 mice per group). **C** Histogram plot of drops number of the right forelimbs in grid walking after photo-activation or photo-inhibition (n = 4 mice per group). Using two-tailed unpaired Student's test. All data are represented as mean ± SD. **D** Representative immunostaining images of DCX^+^ (red) GFP^+^ (green) cells and GFAP^+^ (purple) GFP^+^ (green) cells after NSC-GFP transplantation into glial scar region in sham operated and 3, 7, 14 days mice after ischemic stroke. White arrows indicated co-expression signals. Scale bar = 50 μm. **E** Quantification for proportion of GFP^+^DCX^+^/DCX^+^ and GFP^+^GFAP^+^/GFAP^+^ cells (n = 3 mice per group). Using two-way ANOVA followed by Dunnett's test. **F** and **G** Representative immunostaining images (**F**) and quantification (**G**) of Tuj-1^+^ (green) and GFAP^+^ (red) cells following the differentiation of NSCs in polyacrylamide gels with stiffness ranging from 0.5, 1.0, 10, 20, 45, and 65 kPa. Scale bar = 100 μm (n = 4 slices per group). Using two-way ANOVA followed by Dunnett's test. **H-J** Quantification of total neurite length (**H**), intersection number (**I**) and branches number (**J**) per neuron following NSC differentiation in polyacrylamide gels with varying stiffness values of 0.5, 1.0, 10, 20, 45, and 65 kPa (n = 18 cells per group). One-way ANOVA followed by Dunnett's test.

We determined the effect of modulation of glial scar stiffness on neurological recovery after ischemic stroke by quantifying the numbers of EdU^+^DCX^+^ and EdU^+^CD31^+^ cells in the peri-infarct area of GFAP-ChR2 and GFAP-Arch mice. Optogenetic stimulation of GFAP-Arch mice led to not only a reduction in glial scar stiffness but also the increases of EdU^+^DCX^+^ and EdU^+^CD31^+^ cells, which indicated an increased capacity to promote endogenous neuro-regeneration and angiogenesis for neurological recovery after stroke (**Figure 6A-B and Figure S9B-C**). Furthermore, we characterized SMI31 expression within the glial scar region to evaluate endogenous changes in axonal outgrowth. Quantitative analysis revealed that the photoinhibition group (Cre⁺Arch⁺/⁺light⁺) exhibited a significant increase in SMI31-positive signal compared with controls, suggesting increased axonal regeneration. In contrast, a significant difference in SMI31 signal intensity was not observed between the light-on and light-off groups in the astrocyte activation model (Cre⁺ChR2⁺/⁺), indicating that the light-induced activation of astrocytes did not promote endogenous axonal growth (**Figure S9D-E**). Moreover, beginning on the third day of light stimulation, the mice with ischemic stroke in the photo-inhibition group exhibited an effective reduction in the number of forelimb slips on the injured side during grid walking, suggesting that the neuromotor function improved, whereas no significant difference was detected in the photo-activation group (**Figure 6C**). We confirmed that the change in glial scar stiffness affected the direction of transplanted NSCs differentiation after ischemic stroke by transplanting primary NSCs (**Figure S10A**) isolated from eGFP-mice into the glial scar region at 3, 7 and 14 days after distal MCAO. Although the proportion of transplanted NSCs that differentiated into astrocytes was significantly greater than that of neurons at each time point, the decrease in the proportion of GFP^+^DCX^+^ cells over time suggested that the increased stiffness of glial scar could direct the differentiation of exogenously transplanted NSCs into astrocytes (**Figure 6D-E**).

To further verify transplanted NSCs lineage choices can be modulated by sensing alterations in the stiffness of the glial scar microenvironment, we developed polyacrylamide hydrogels that mimicked the glial scar stiffness at different time points following ischemic stroke (**Figure S10B-C**). When NSCs were cultured in the hydrogel conditions at 0.5, 1.0, 10, 20, 45, and 65 kPa, the proportion of Tuj-1^+^ cells decreased while the proportion of GFAP^+^ cells increased with increasing stiffness, which suggested that softer the hydrogel was, the more favorable it was for the differentiation of NSCs into neurons. Specifically, a hydrogel with a stiffness of 1.0 kPa was found to be optimal, as it closely resembled the stiffness of normal brain tissue (**Figure 6F-G**). Remarkably, the stiffer hydrogel significantly decreased the total branch length per neuron, as well as the numbers of branches and intersections, indicating that the softer microenvironment also increased the complexity of differentiated neurons (**Figure 6H-J and Figure S10G**). The sensory response of NSCs to hydrogel stiffness had a threshold between 1.0 kPa and 10 kPa, beyond which their neuronal differentiation ability and complexity dropped abruptly. However, this pattern did not appear to the proliferation capacity of NSCs (**Figure S10D-F**).

Overall, changes in the proportion of Wnt7b^+^ astrocytes caused by optogenetic stimulation can modulate the stiffness of glial scar tissue following stroke, softening the microenvironment, facilitating both endogenous and exogenous NSC differentiation into neurons, and subsequently enhancing the recovery of neuromotor function after stroke.

## Discussion

Understanding how the mechanical properties of reactive astrocytes affect microenvironment stiffness is crucial for neuro-regeneration after brain injury. In this study, our results support the hypothesis that a morphological transition in astrocytes changes glial scar stiffness, which affects neurological recovery after stroke. First, using AFM *in vitro* and USE *in vivo,* the peri-infarct area was observed to become stiffer during glial scar formation. Second, the increase in glial scar stiffness was attributed primarily to an upregulation of Wnt7b^+^ astrocytes. This process was facilitated by Piezo1, which activated the noncanonical Wnt7b-Ca^2+^ signaling pathway and subsequently induced cytoskeletal reorganization. Third, modulating glial scar stiffness affected the lineage choice during both endogenous neuro-regeneration and exogenous NSC differentiation. Overall, our study sheds light on the complex interplay among astrocytic activation, glial scar stiffness, and neuro-regeneration, highlighting the potential of Piezo1 as a therapeutic target to manipulate cellular responses to mechanical cues in stroke recovery.

The mechanical properties of the microenvironment have important implications for cellular development, differentiation and regeneration 43. Although a previous study has indicated that scar tissue in the rat brain cortex becomes softer after traumatic injury than surrounding normal tissue does 20, our investigation systematically supports the hypothesis that glial scars in the peri-infarct area are actually stiffer than the tissue under normal conditions, according to multidimensional measurements. First, this discrepancy may stem from the use of different animal models or the increased precision of our smaller-diameter microspheres (12 μm), which allow more focused targeting at the single-cell level than the 89.3 μm polystyrene beads. Therefore, the microinjuries generated by the needling model, when subjected to the 89.3 μm beads for the indentation test, may primarily assess damage to the needle tract rather than the glial scar region. Second, our AFM data revealed a greater Young's modulus in the glial scar area following ischemia than in normal tissue. Third, recognizing the limitations of AFM when applied to *in vitro* tissue due to potential autolysis effects, we further used USE for *in vivo* analysis, which demonstrated an increase in Young's modulus of glial scar area during ischemia process. Together, these findings underscore the significant changes in tissue stiffness associated with ischemic stroke-induced glial scar formation, highlighting the importance of considering both *in vitro* and *in vivo* approaches to accurately characterize and understand the mechanical properties of scar tissue under neurological conditions.

Mechanosensitive ion channels, such as Piezo ion channels, are essential for detecting external mechanical stimuli and sensing mechanical forces within tissues. Astrocytes can use Piezo1 to explore local mechanical cues and actively generate localized Ca^2+^ signaling 26. Our research indicated that Piezo1 in astrocytes responds to mechanical stimuli by regulating the cytoskeleton to alter its stiffness. Ca²⁺ signaling may play a crucial role in this process. Wnt signaling has been shown to target the cytoskeleton and regulate cell morphology 16, 44, 45.

Both siRNA-mediated knockdown of Piezo1 and Yoda1 treatment significantly modulated intracellular Ca^2+^ levels, Wnt7b expression, and cytoskeletal reorganization, leading to alterations in astrocyte morphology *in vitro*. This phenomenon may be attributed to the disruption of calcium influx homeostasis across the cell membrane following Piezo1 suppression, which subsequently triggers the release of additional free calcium ions from intracellular endoplasmic reticulum stores in response to the stimulus. Although both Wnt-PCP and Wnt-Ca^2+^ pathway are involved in this process, our results indicated that Wnt-Ca^2+^ signaling plays a major role in Piezo1 mediated regulation. However, how intracellular calcium is released after Piezo1 knockdown needs to be explored in the future. Furthermore, optogenetic regulation alters Wnt7b expression and glial scar stiffness by altering Ca^2+^ levels in astrocytes. Spatially resolved proteomics also supports changes in cytoskeletal reorganization in fibrotic astrocytes (Wnt7b^+^GFAP^+^ cells). The ECM also contributes to tissue stiffness 46, 47, and discrepancies in matrix organization are attributed to different astrocyte types 48. In particular, the GO/KEGG functional enrichment analyses of *in vitro* RNA-sequencing data revealed that the top-ranked terms were also associated with ECM-related pathways, such as ECM-receptor interaction, collagen binding, and integrin binding. Notably, genes encoding ECM components, including members of the collagen, filamin, and laminin families, were significantly downregulated in fibrotic astrocytes. These findings suggest that ECM deposition may likewise contribute to the morphological alterations and stiffness of astrocytes. However, our results showed a weak correlation between the ECM component and glial scar stiffness. The reason could be that variances in astrocyte stiffness themselves and cytoskeletal rearrangements cause local alignment of the surrounding nonlinearly elastic ECM components, creating a positive mechanical feedback loop between these components and the fibrosis of astrocytes 49.

During CNS regeneration after ischemic stroke, stem cells and growing axons interact mechanically with a complex microenvironment, including variations in tissue stiffness 50, 51. Differences in tissue stiffness strongly influence the lineage specification and commitment of stem cells, which is critical for regenerative medicine and stem cell therapy 27, 51, 52. Our previous study demonstrated that most transplanted NSCs differentiate into astrocytes after ischemic stroke 31, indicating that the stiffness of glial scar may direct the NSC lineage choice during the recovery stage of ischemic stroke. In this study, by using poly-acrylamide hydrogels to mimic the stiffness of the glial scar at various post-stroke time points, we observed that softer microenvironment induced the differentiation of NSCs into neurons, which promoted the recovery of neuromotor function after stroke.

Overall, our study indicated that modulating Piezo ion channel activity could be a valuable approach for regulating glial scar stiffness, which can subsequently direct the stem cell lineage choice. In future regenerative medicine and stem cell transplantation strategies, the integration of appropriate mechanical signals, in addition to pharmacological and cellular therapies, should be considered to optimize functional recovery following ischemic stroke.

## Supplementary Material

Supplementary figures and tables.
