# Supplementary material for: Piezo1 induces Wnt7b+ astrocytes transformation to modulate glial scar stiffness and neuro-regeneration after stroke
Source: Theranostics. 2026 Jan 1;16(2):668–88. doi: 10.7150/thno.120838 (PMC12674932; doi:10.7150/thno.120838)
Supplement: Supplementary file 1 — Supplementary figures and tables. [file thnov16p0668s1.pdf]

Supplementary Materials for

Piezo1 Induces Wnt7b$^+$ Astrocytes Transformation to Modulate Glial Scar Stiffness and Neuro-regeneration after Stroke

Shengju Wu *et al.*
*Corresponding author. Email: gyyang@sjtu.edu.cn

This PDF file includes:

Figures. S1 to S10
Legend for Figures. S1 to S10
Tables S1 to S2

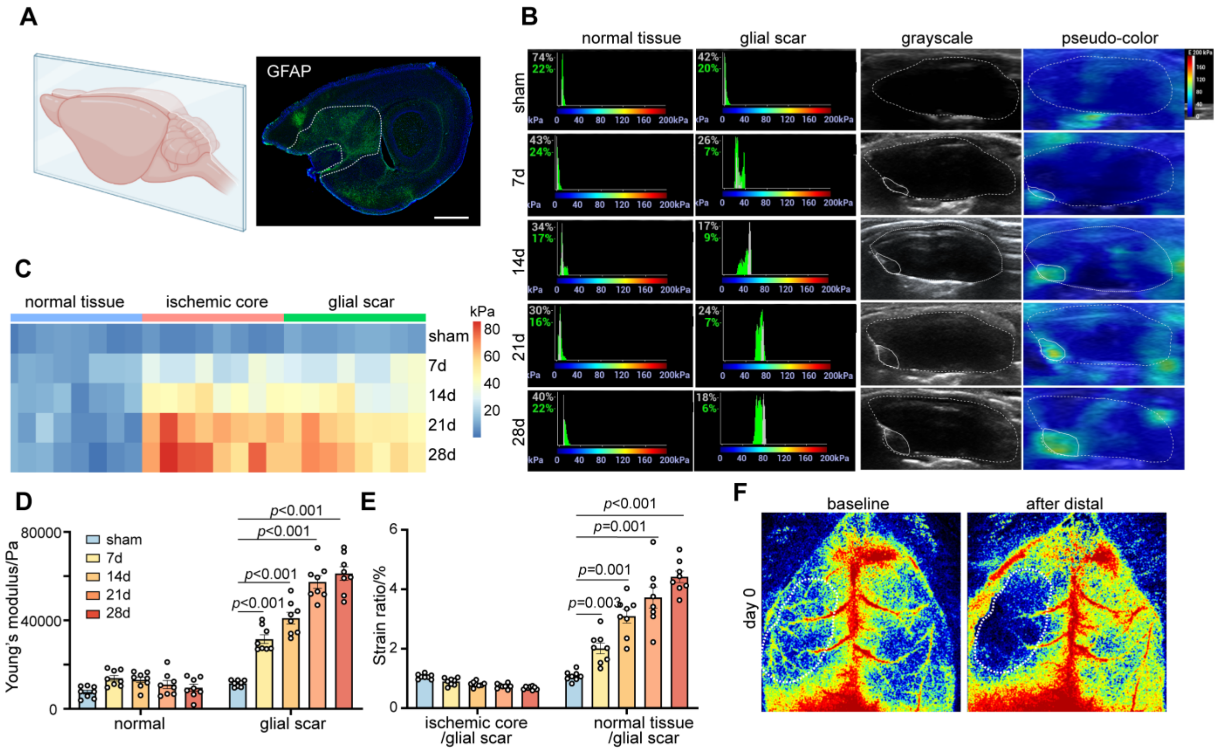

Figure. S1: The stiffness of glial scar increases with time after transient middle cerebral artery occlusion.

A Schematic diagram of brain slices from mice subjected transient middle cerebral artery occlusion (MCAO). The fluorescent imaging represents the sagittal plane. Scale bar = 1 mm. **B** Representative images of stiffness detected by Ultrasound elastography at the sham operated, and 7, 14, 21, 28 days mice after transient MCAO in normal tissue and glial scar, highlighting the glial scar region in grayscale (left column) and pseudo-color image (right column). Solid white lines represent brain parenchyma and glial scar outlines. The left panel displays the percentage of the maximum possible stiffness value within the hardness range of the target area (The gray percentage represents the center area of the ROI, while the green percentage represents the shell of the ROI). **C** Heat map of the normal regions, ischemic core, and glial scar stiffness in sham, and 7, 14, 21, 28 days after transient MCAO. **D** and **E** Statistical analysis showing changes in Young's modulus

(**D**) and strain ratio (**E**) over time after transient MCAO (n = 8 mice per group). **F** Representative images of mice cerebral blood flow measured by Laser speckle contrast imaging (LSCI) at the baseline and 10 min after distal MCAO. Using one-way ANOVA followed by Dunnett's test (**D**, **E**). Data compared to the sham operated mice. All data are represented as mean ± SD.

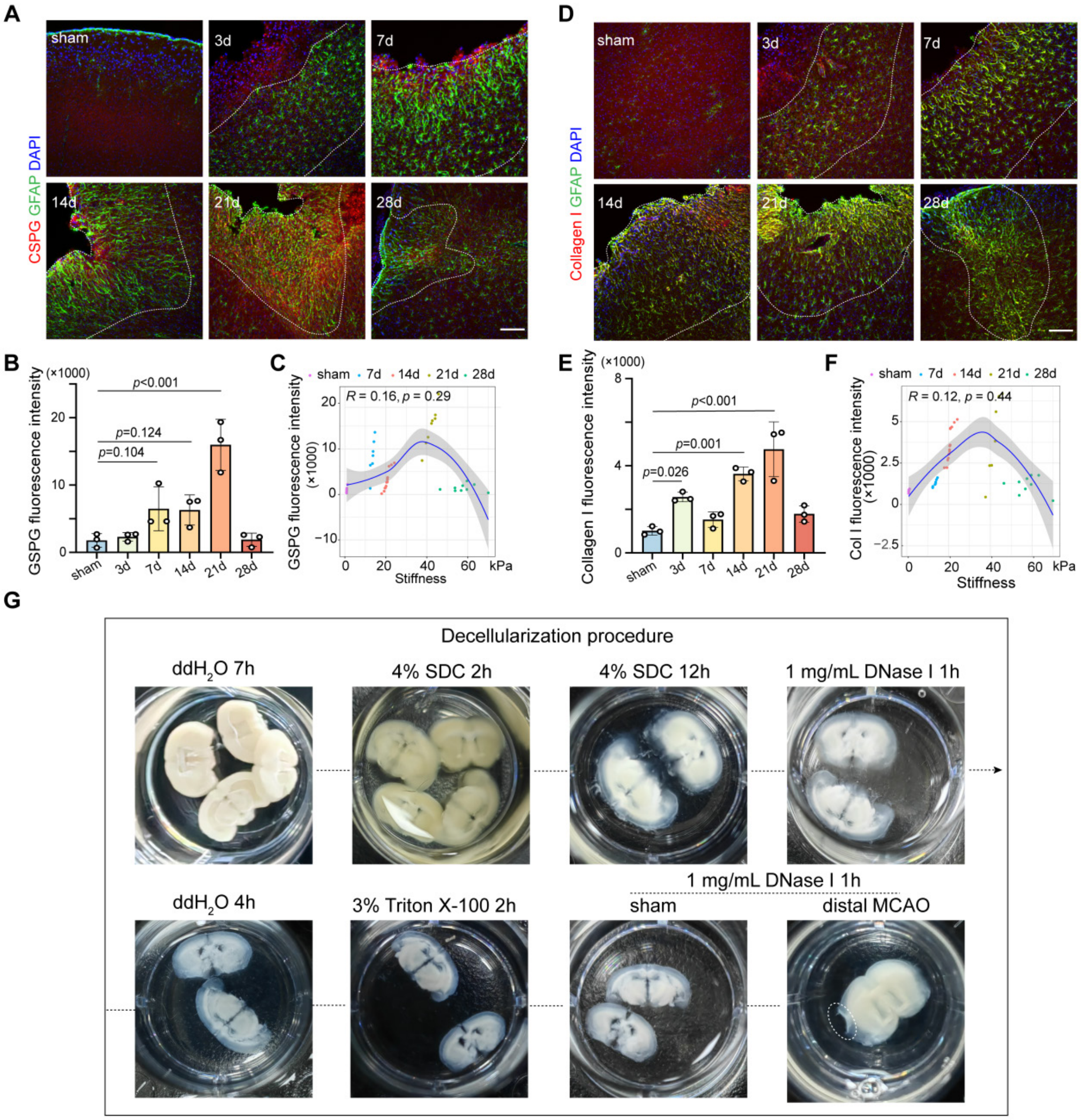

Figure. S2: Glial scar stiffness is weakly correlated with extracellular matrix components after distal MCAO.

A Representative immunostaining images of chondroitin sulfate proteoglycan (CSPG; red), glial fibrillary acidic protein (GFAP; green) and DAPI (blue) in the glial scar region at different time points after distal MCAO. Scale bar = 100 μm. **B** Quantification of CSPG fluorescence intensity in sham operated and 3, 7, 14, 21, 28 days after distal MCAO mice (n = 3 mice per group). **C** Correlation of glial scar stiffness with CSPG (R = 0.16, $p = 0.29$) fluorescence intensity over time following distal MCAO. **D** Representative images of Collagen I (red), GFAP (green) and DAPI (blue) in the glial scar at different time points after distal MCAO. Scale bar = 100 μm. **E** Quantification of Collagen I fluorescence intensity in sham operated and 3, 7, 14, 21, 28 days after

distal MCAO mice (n = 3 mice per group). **F** Correlation of glial scar stiffness with Collagen I ($R = 0.12$, $p = 0.44$) fluorescence intensity over time following distal MCAO. **G** Decellularization procedure. Representative images for shape, size and color of the 2.0 mm mouse brain section during the whole decellularization procedure. ddH2O: demineralized water, SDC: sodium deoxycholate.

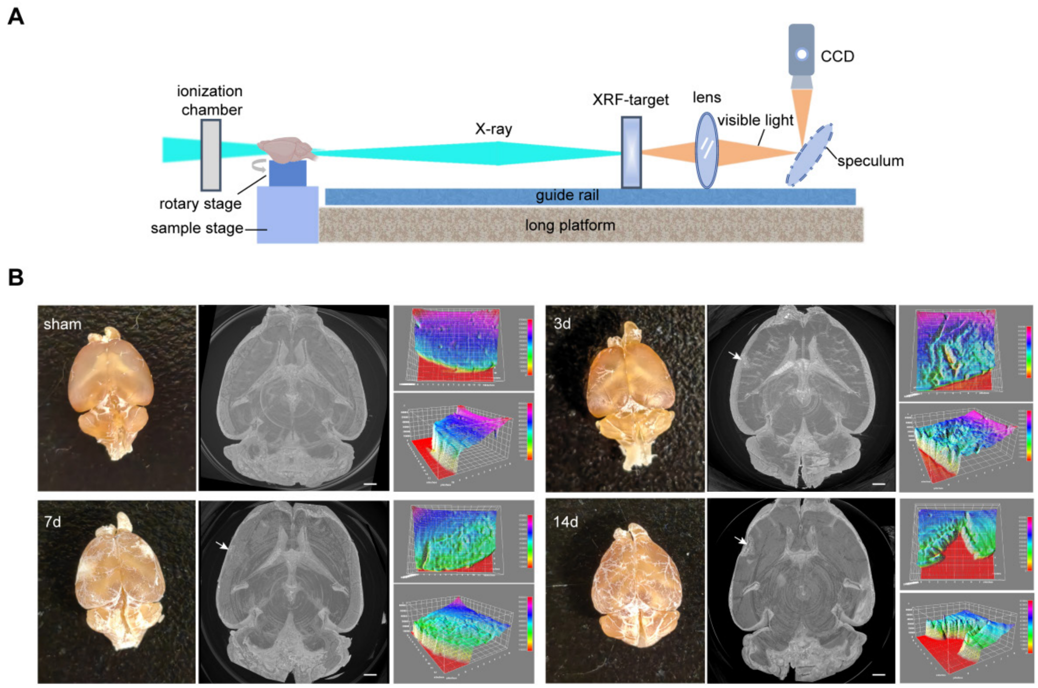

Figure. S3: Synchrotron radiation phase-contrast imaging of glial scar after distal MCAO.

A Schematic diagram of the operation at the BL13HB beamline at the Shanghai Synchrotron Radiation Facility. **B** Schematic representation of sham and 3, 7, 14 days after distal MCAO, white arrows indicating glial scar region. Scale bar = 500 μm.

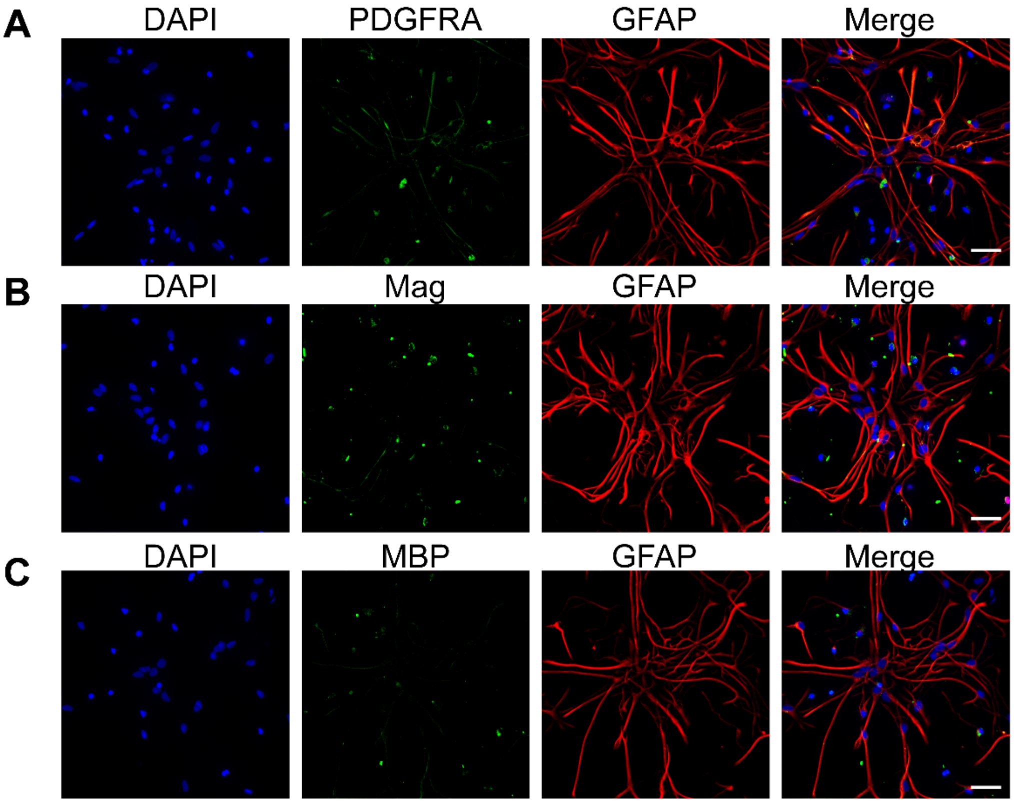

Figure. S4: **The markers of OPCs show low co-expression with fibrotic astrocytes.**

A representative immunofluorescence image showing co-labeling of PDGFRA (green) and GFAP (red) in fibrotic astrocytes, scale bar = 100 µm. **B** representative immunofluorescence image showing co-labeling of Mag (green) and GFAP (red) in fibrotic astrocytes, scale bar = 100 µm. **C** representative immunofluorescence image showing co-labeling of MBP (green) and GFAP (red) in fibrotic astrocytes, scale bar = 100 µm.

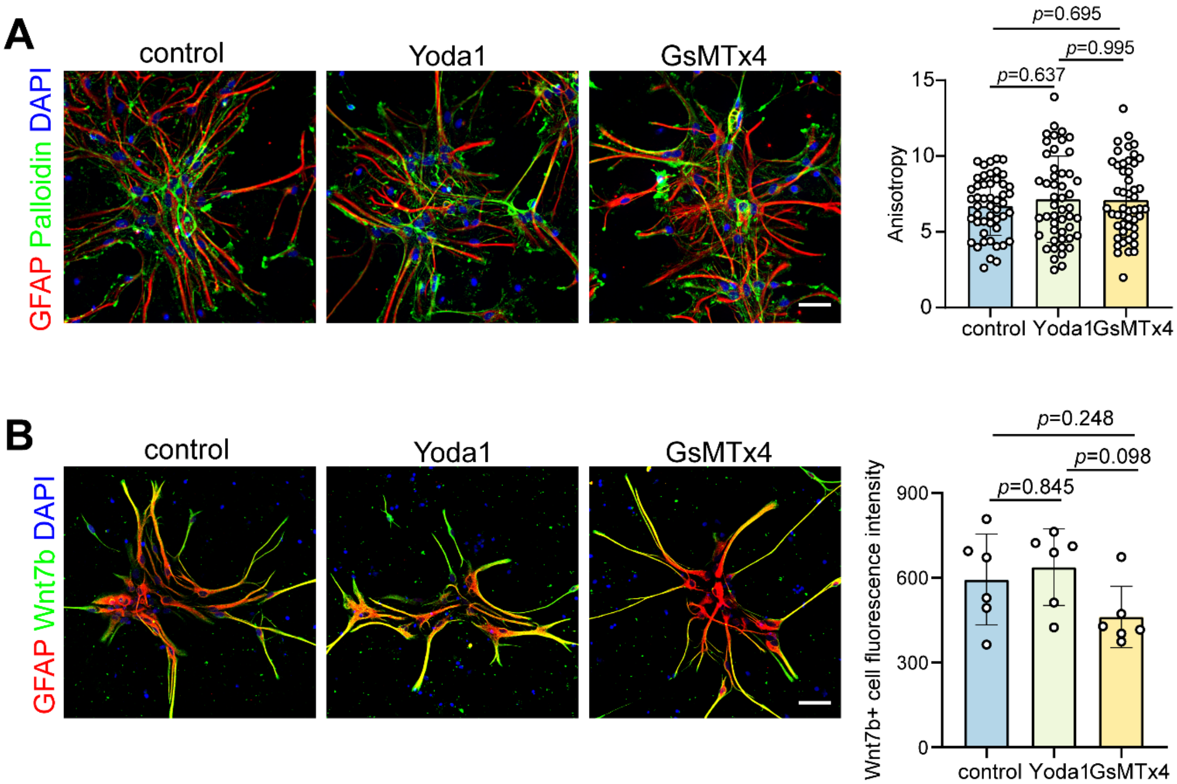

Figure. S5: The activation or inhibition of Piezo1 does not alter the morphology of Wnt7b^{+} fibrotic astrocytes.

A Representative immunostaining images of Wnt7b^{+} astrocytes with the Piezo1 activator Yoda1 (5 μM) and inhibitor GsMTx4 (2.5 μM) for 24 h compared to the DMSO control group. Phalloidin (green) and GFAP (red) with quantitative anisotropy analysis. Scale bar = 100 μm (n = 49 cells).
B Representative immunostaining images of Wnt7b (green) in Wnt7b^{+} astrocytes (GFAP, red) following activation of Piezo1 activity by Yoda1 and GsMTx4. Scale bar = 100 μm. And quantification of Wnt7b fluorescence intensity in astrocytes (n = 6 slices per group). One-way ANOVA followed by Dunnett’s test. All data are represented as mean ± SD.

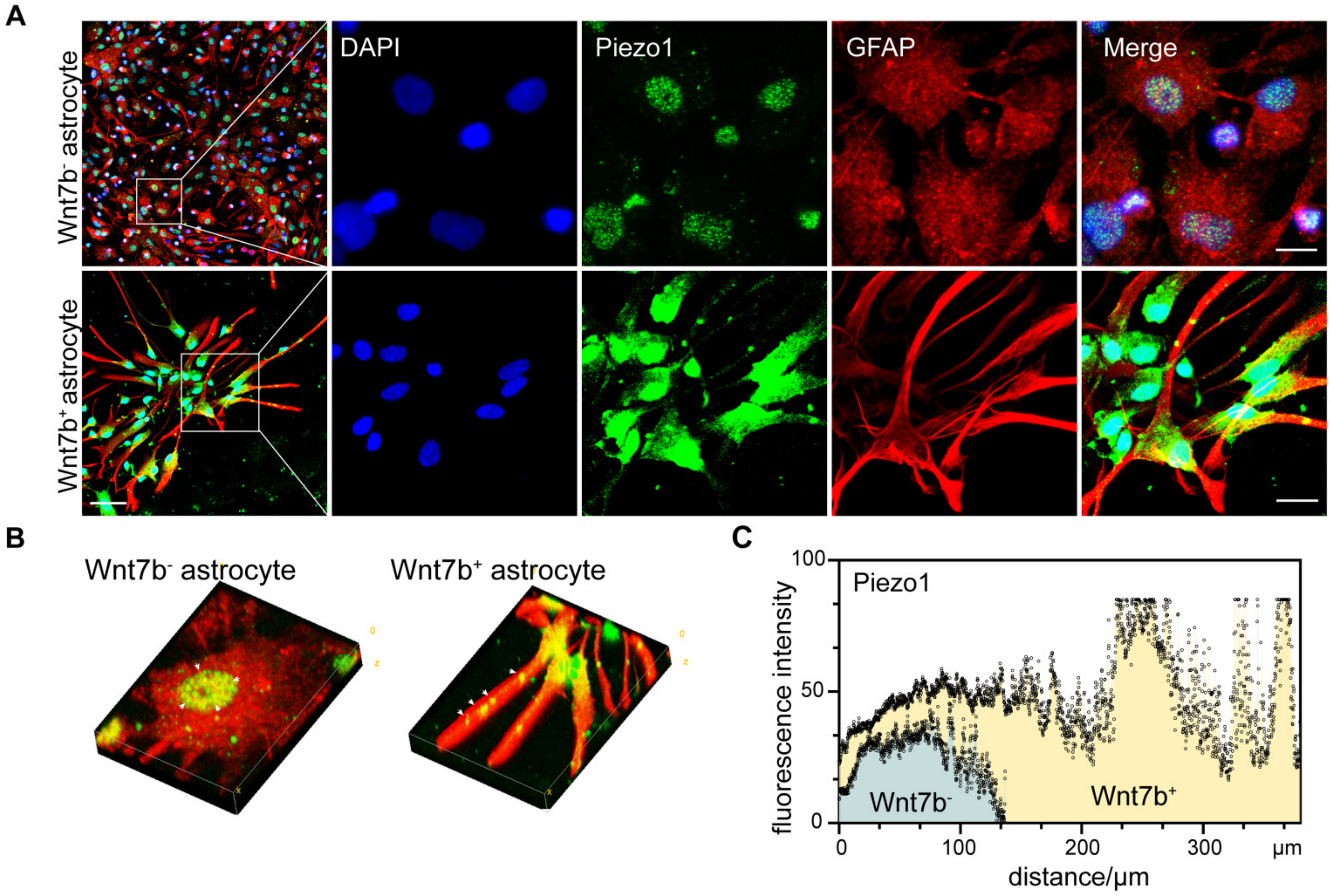

Figure. S6: Distribution of Piezo1 in normal and Wnt7b^{+} astrocyte.

A Representative immunostaining images showing Piezo1 (green) distribution in primary normal and Wnt7b^{+} astrocytes culture, co-stained with (GFAP, red). Scale bar = 50 μm. Scale bar = 10 μm in Wnt7b^{-} astrocytes and scale bar = 20 μm in Wnt7b^{+} astrocytes. **B** 3D schematic diagram and quantitation of Piezo1 distribution in individual normal and Wnt7b^{+} astrocytes. White arrow heads indicated Piezo1 expression region. **C** Statistical analysis of fluorescence intensity comparing normal and Wnt7b^{+} astrocytes.

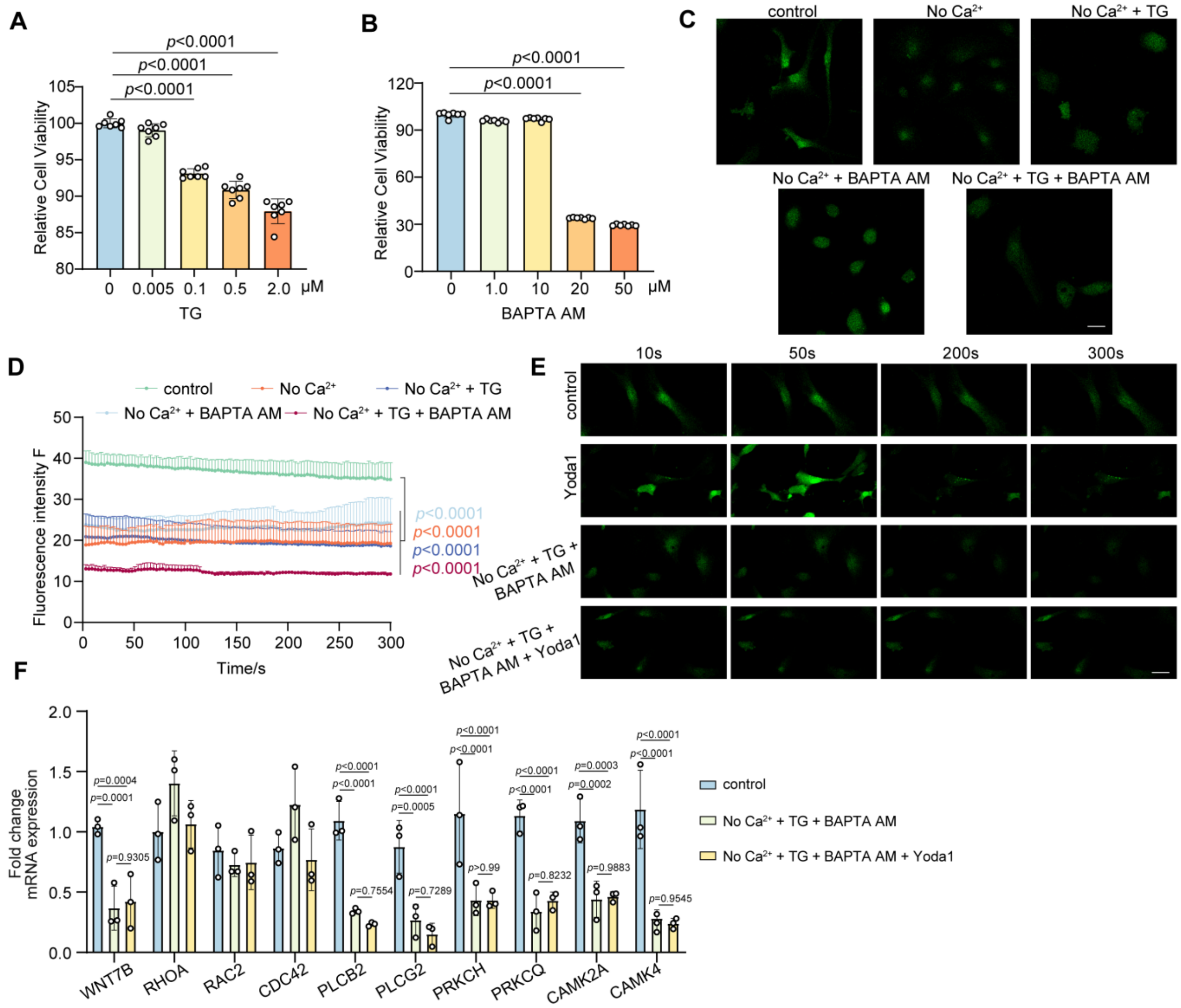

Figure. S7: Inhibition of astrocyte calcium flow significantly reduced Wnt signaling pathway gene expression.

A Quantification of astrocyte viability normalized to 0 μM when different concentration thapsigargin (TG) treatment (n = 7 wells per group). **B** Quantification of astrocyte viability normalized to 0 μM when different concentration 1, 2-Bis (2-aminophenoxy) ethane-N, N, N', N'-tetraacetic acid acetoxymethyl ester (BAPTA AM) treatment (n = 7 wells per group). **C** Representative immunostaining images of Ca^{2+} imaging in Fluo-4 AM after control (DMSO), Ca^{2+}-free DMEM, Ca^{2+}-free DMEM + TG (5 nM), Ca^{2+}-free DMEM + BAPTA AM (10 μM) and Ca^{2+}-free DMEM + TG (5 nM) + BAPTA AM (10 μM) treatment in astrocyte culture. Scale bar =

100 μm. **D** Quantification of fluorescence intensity F representing the real-time Ca^{2+} signal in different treatment groups (Same groups as in panel **C**). **E** Representative immunostaining images of Ca^{2+} imaging in Fluo-4 AM after control (DMSO), control + Yoda1 (5μM), Ca^{2+}-free DMEM + TG (5 nM) + BAPTA AM (10 μM) and Ca^{2+}-free DMEM + TG (5 nM) + BAPTA AM (10 μM) + Yoda1 (5 μM) treatment in astrocyte culture. Scale bar = 100 μm. **F** Real-time PCR analysis of Wnt pathway related genes in control, Ca^{2+}-free DMEM + TG (5 nM) + BAPTA AM (10 μM) and Ca^{2+}-free DMEM + TG (5 nM) + BAPTA AM (10 μM) + Yoda1 (5 μM) treatment groups (n = 3 independent primary astrocytes cultures). GAPDH was used as an internal control.

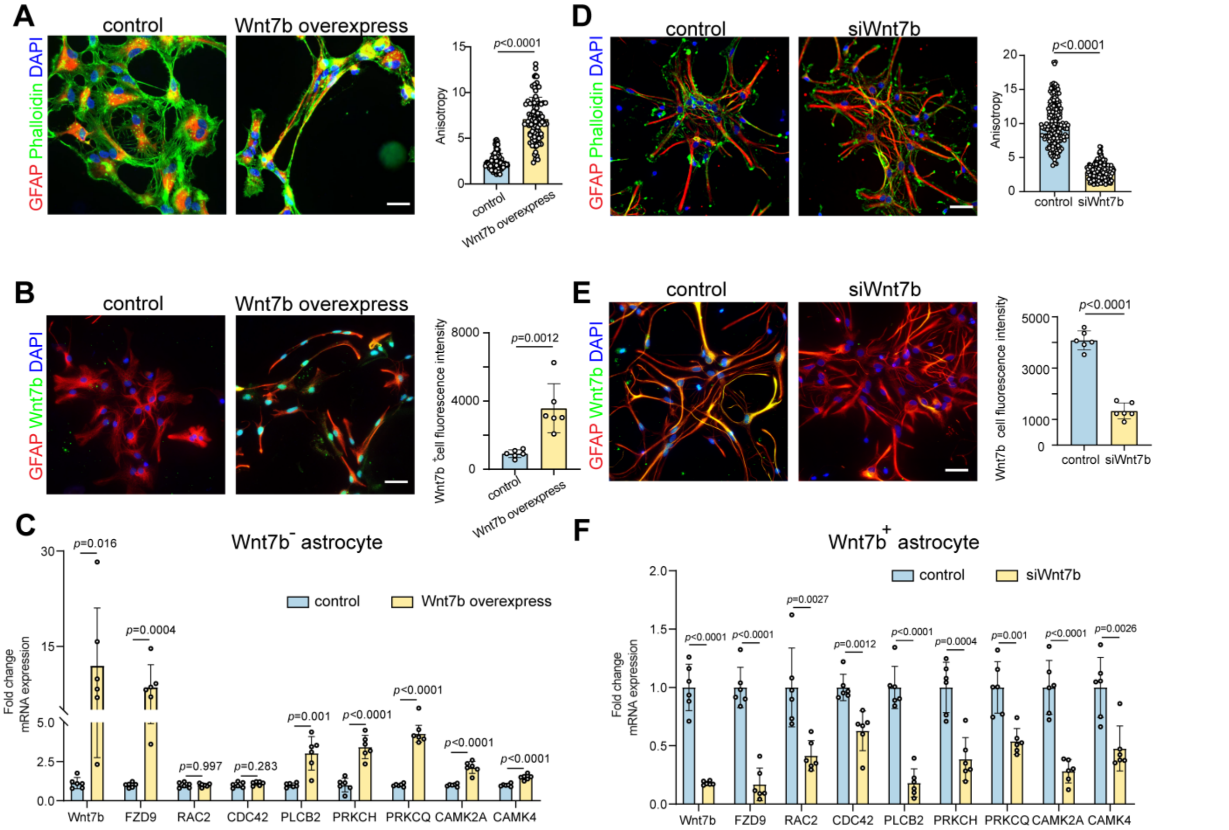

Figure. S8: Wnt7b regulates astrocytic fibrosis through the Wnt-Ca^{2+} signaling pathway.

A Representative immunostaining images showing overexpression of Wnt7b in $Wnt7b^-$ astrocytes and quantitative analysis of anisotropy in control and Wnt7b over expression groups (Wnt7b over-exp.) *in vitro*. Scale bar = 50 µm. Phalloidin (green) and GFAP (red) visualization followed (n = 88 cells). **B** Representative immunostaining images of Wnt7b (green) in $Wnt7b^-$ astrocytes (GFAP, red) following Wnt7b overexpression. Scale bar = 50 µm. And quantification of fluorescence intensity of Wnt7b in astrocytes (n = 6 slices per group). **C** Real-time PCR analysis of Wnt pathway related genes between control and Wnt7b overexpression in $Wnt7b^-$ astrocytes (n = 6 independent primary astrocytes cultures). GAPDH was used as an internal control. **D** Representative immunostaining images showing siRNA of Wnt7b in $Wnt7b^+$ astrocytes and quantitative analysis of anisotropy in control and Wnt7b siRNA groups (siWnt7b) *in vitro*. Scale bar = 50 µm. Phalloidin (green) and GFAP (red) visualization followed (n = 171

cells). **E** Representative immunostaining images of Wnt7b (green) in Wnt7b$^+$ astrocytes (GFAP, red) following Wnt7b siRNA knockdown. Scale bar = 50 μm. And quantification of fluorescence intensity of Wnt7b in Wnt7b$^+$ astrocytes (n = 6 slices per group). **F** Real-time PCR analysis of Wnt pathway related genes between control and Wnt7b siRNA knockdown in Wnt7b$^+$ astrocytes (n = 6 independent primary astrocytes cultures). GAPDH was used as an internal control. Using T-test and all data are represented as mean ± SD. One-way ANOVA followed by Dunnett's test.

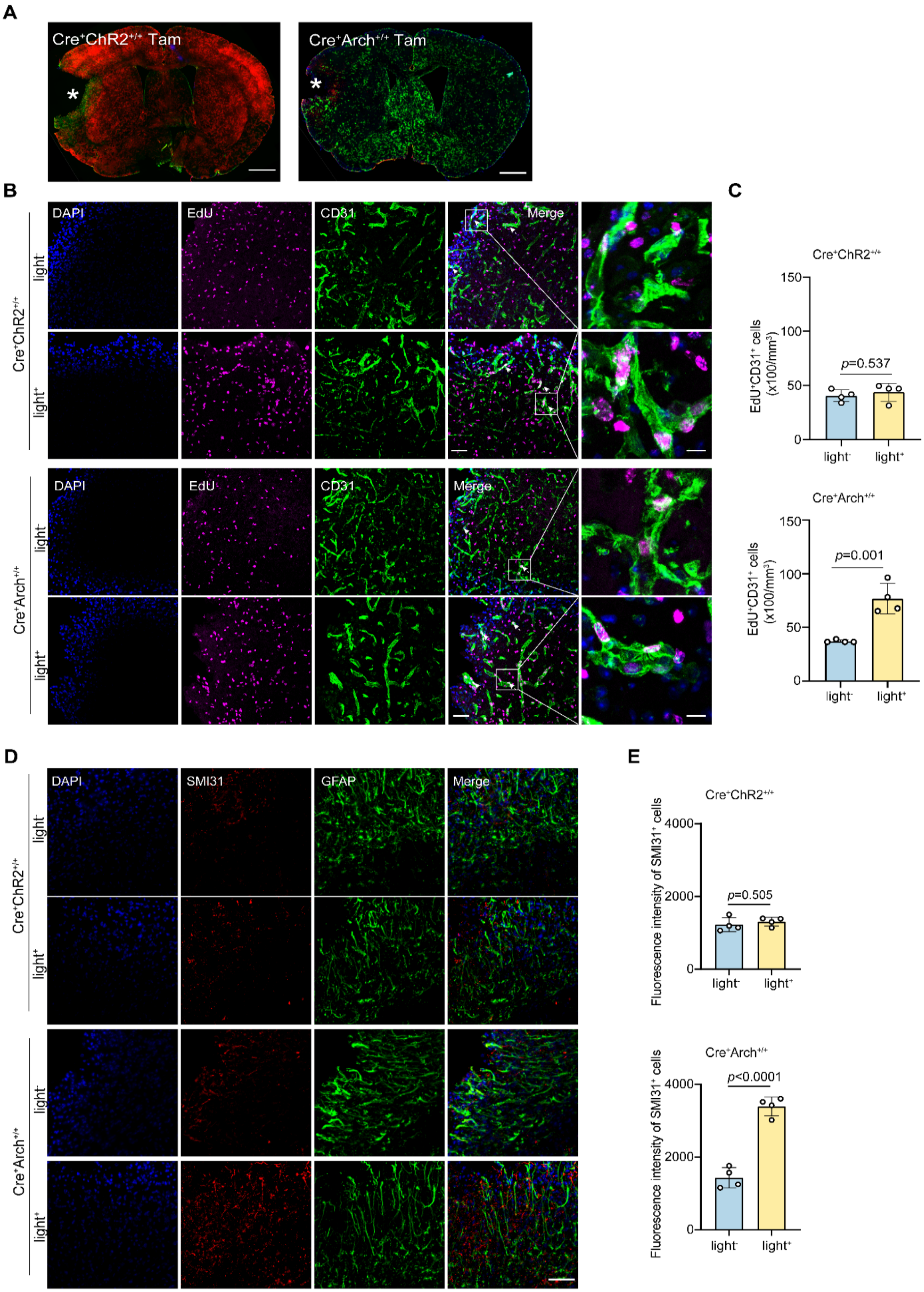
A
Cre+ChR2+/+ Tam
Cre+Arch+/+ Tam
B
DAPI
EdU
CD31
Merge
light-
light+
Cre+ChR2+/+
Cre+Arch+/+
C
Cre+ChR2+/+
EdU+CD31+ cells (x100/mm3)
p=0.537
p=0.001
Cre+Arch+/+
D
SMI31
GFAP
E
Fluorescence intensity of SMI31+ cells
p=0.505
p<0.0001

142

Figure. S9: Photoinhibition in astrocytes of glial scar region promotes angiogenesis after stroke.

A Coronal section of fluorescence expression efficiency after ChR2 activation and Arch inhibition with tamoxifen (Tam) treatment. The stars indicate the region of damage. Scale bar = 1 mm. **B** Representative immunostaining images demonstrating EdU^+ (purple) $CD31^+$ (green) following ChR2 photo-activation or Arch photo-inhibition in mice. Scale bar = 50 µm. **C** Quantification of EdU^+CD31^+ cell number in ChR2 photo-activation or Arch photo-inhibition mice (n = 5 mice in $Cre^+ChR2^{+/+}$ group and n = 4 mice in $Cre^+Arch^{+/+}$ group). **D** Representative immunostaining images demonstrating $SMI31^+$ (red) $GFAP^+$ (green) following ChR2 photo-activation or Arch photo-inhibition in mice. Scale bar = 50 µm. **E** Quantification of $SMI31^+$ fluorescence intensity following ChR2 photo-activation or Arch photo-inhibition mice (n = 4 mice in $Cre^+ChR2^{+/+}$ group and n = 4 mice in $Cre^+Arch^{+/+}$ group). Using two-tailed unpaired Student's test. All data are represented as mean ± SD.

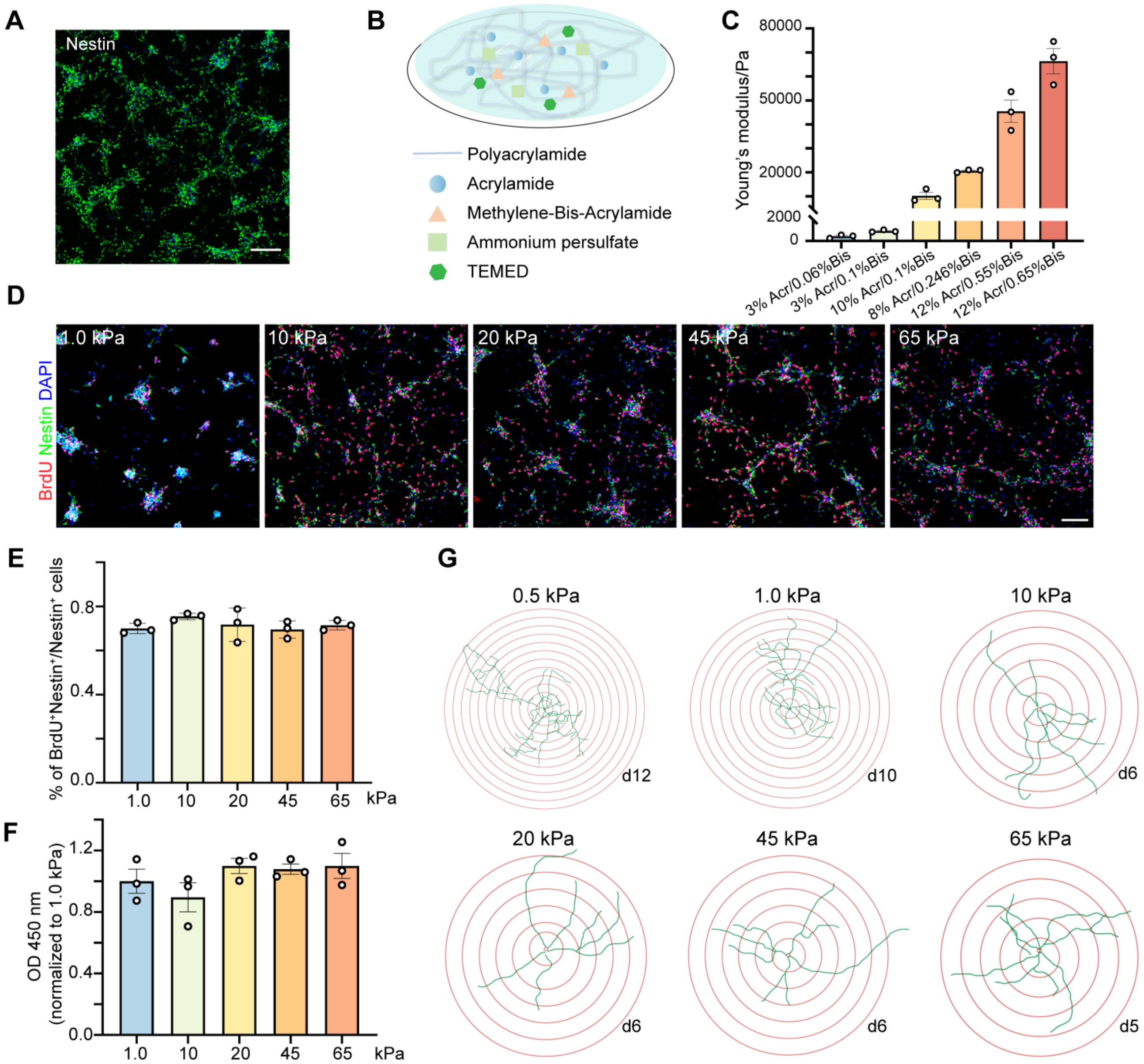

Figure. S10: The niche stiffness has no effect on NSC proliferation.

A Representative immunostaining image of Nestin (green) to assess purity of primary isolated NSCs. Scale bar = 100 μm. **B** Schematic diagram of the composition of polyacrylamide gels preparation. **C** AFM measured the Young's modulus for different ratios of Acr (Acrylamide) and Bis (Methylene-Bis-Acrylamide). **D** Representative immunostaining images of $BrdU^+$ (red) $Nestin^+$ (green) NSCs in polyacrylamide gels at 1.0, 10, 20, 45 and 65 kPa. Scale bar = 100 μm.

E Quantification of $BrdU^+Nestin^+/Nestin^+$ cells rate (n = 3 slices per group). **F** Quantification of OD 450 nm normalized to 1.0 kPa (n = 3 slices per group). **G** Schematic representation illustrating the complexity of single neuron intersection number. $\Delta d = 50$ μm. Using one-way ANOVA followed by Dunnett's test (**C**, **E**, **F**). All data are represented as mean ± SD.

Table S1.

Table S1. Acrylamide gel concentration

Arc %	Bis %	40%Arc /[μl]	2%Bis/ [μl]	1M HEPES/[μl]	ddH_2O/[μl]	10% APS/ [μl]	TEMED /[μl]	Theoretical Ey/[kPa]	Practical Ey/[kPa]
3	0.06	75	30	10	874	10	1	0.5	0.46
3	0.1	75	50	10	854	10	1	1.0	0.99
5	0.15	125	75	10	779	10	1	5.0	5.84
10	0.1	250	50	10	679	10	1	10.0	10.18
12	0.145	300	72.5	10	606.5	10	1	15.0	31.01
8	0.246	200	123	10	656	10	1	20.0	20.60
10	0.225	250	112.5	10	616.5	10	1	25.0	24.38
12	0.28	300	140	10	539	10	1	30	11.07
10	0.3	250	150	10	579	10	1	35	27.71
8	0.48	200	240	10	539	10	1	40	15.93
12	0.55	300	275	10	404	10	1	45	45.43
12	0.575	300	287.5	10	391.5	10	1	50	60.76
12	0.6	300	300	10	379	10	1	55	53.27
12	0.625	300	312.5	10	366.5	10	1	60	51.27
12	0.65	300	325	10	354	10	1	65	66.518
12	0.675	300	337.5	10	341.5	10	1	70	68.219

TableS2. Table S2. Sequence of primers

Genes	Forward sequence (5'-3')	Reverse sequence (5'-3')
Piezo1	TTCCTGCTGTACCAGTACCT	AGGTACAGCCACTTGATGAG
Piezo2	CACCTGGCTACAACTGCTCA	CCCGATGTCAGGTACAAACA
Yap1	AGGAGAGACTGCGGTTGAAA	CCCAGGAGAAGACACTGCAT
Trpc4	GCTGGAGGAGAAGACACTGG	GACCTGTCGATGTGCTGAGA
Trpc6	CAGGCCCAGATTGATAAGGA	CCAGCTTTGGCTCTAACGAC
Trpm2	TGGATCATGAGTGTGCAGGT	ACAGACAATGCCTGGATCG
Trpm6	GACGTTCATAGTGGACCTCTC	GTTGATCAGCATGTCTCTGTTC
Trpm7	CCTCATGAAGACCATTTTCTAA	ACAACTGTAACCTTCCTCACAG
Trpa1	GCAGGTGGAACTTCATACCAACT	CACTTTGCGTAAGTACCAGAGTGG
Trpv2	TGATGAAGGCTGTGCTGAAC	CACCACAGGCTCCTCTTCTC
Trpv4	ACAACACCCGAGAGAACACC	TGAACTTGCGAGACAGATGC
Wnt7b	TGTCAGGCTCCTGTACCACC	AGTTGGGCGACTTCTCGATG
RHOA	AGCCTGTGGAAAGACATGCTT	TCAAACACTGTGGGCACATAC
RAC2	CGTCAGCCCAGCCTCTTATG	TCAGGCCTCTCTGGGTGAG
CDC42	CCATCGGAATATGTACCGACTG	CTCAGCGGTCGTAATCTGTCA
PLCB2	ATGGAGTTCCTGGATGTCACG	CGGAGTTTCTGGCTCTTGGG
PLCG2	CGAGGCGATGTGGATGTCAA	AGTGCCGAGTCCATTTCTGG
PRKCH	TCCGGCACGATGAAGTTCAAT	TACGCTCACCGTCAGGTAGG

PRKCQ	GGACTCAAGTGTGATGCATGTGG	TAAGCAGCGAGCCTGTTGAGTG
CAMK2A	CATGGTTTGGGTTTGCAGGG	CCGGCTTTGATCTGCTGGTA
CAMK4	ATCCACCTTCAACCCAA	GATCCTGAGGCACCATAC
GAPDH	GGTGTGAACCATGAGAAGTATGA	GAGTCCTTCCACGATACCAAAG
